# Regional, circuit and network heterogeneity of brain abnormalities in psychiatric disorders

**DOI:** 10.1038/s41593-023-01404-6

**Published:** 2023-08-14

**Authors:** Ashlea Segal, Linden Parkes, Kevin Aquino, Seyed Mostafa Kia, Thomas Wolfers, Barbara Franke, Martine Hoogman, Christian F. Beckmann, Lars T. Westlye, Ole A. Andreassen, Andrew Zalesky, Ben J. Harrison, Christopher G. Davey, Carles Soriano-Mas, Narcís Cardoner, Jeggan Tiego, Murat Yücel, Leah Braganza, Chao Suo, Michael Berk, Sue Cotton, Mark A. Bellgrove, Andre F. Marquand, Alex Fornito

**Affiliations:** 1 Turner Institute for Brain and Mental Health, School of Psychological Sciences, Monash University, Melbourne, Victoria Australia; 2 Monash Biomedical Imaging, Monash University, Melbourne, Victoria Australia; 3 Department of Bioengineering, School of Engineering and Applied Science, University of Pennsylvania, Philadelphia, PA USA; 4 Department of Psychiatry, Rutgers University, Piscataway, NJ USA; 5 School of Physics, University of Sydney, Sydney, New South Wales Australia; 6 BrainKey Inc, Palo alto, CA USA; 7 Donders Centre for Cognitive Neuroimaging, Radboud University, Nijmegen, the Netherlands; 8 Donders Institute for Brain, Cognition and Behaviour, Radboud University, Nijmegen, the Netherlands; 9 Department of Psychiatry, University Medical Center Utrecht, Utrecht, the Netherlands; 10 Department of Cognitive Science and Artificial Intelligence, Tilburg University, Tilburg, the Netherlands; 11 Norwegian Centre for Mental Disorders Research, Division of Mental Health and Addiction, University of Oslo and Oslo University Hospital, Oslo, Norway; 12 Department of Psychiatry and Psychotherapy, Tübingen Center for Mental Health (TÜCMH), University of Tübingen, Tübingen, Germany; 13 Department of Psychiatry, Donders Institute of Brain, Cognition and Behaviour, Radboud University Medical Center, Nijmegen, the Netherlands; 14 Department of Human Genetics, Donders Institute of Brain, Cognition and Behaviour, Radboud University Medical Center, Nijmegen, the Netherlands; 15 Department of Cognitive Neuroscience, Radboud University Medical Centre, Nijmegen, the Netherlands; 16 Centre for Functional MRI of the Brain, Nuffield Department of Clinical Neurosciences, Wellcome Centre for Integrative Neuroimaging, University of Oxford, Oxford, UK; 17 Department of Psychology, University of Oslo, Oslo, Norway; 18 KG Jebsen Centre for Neurodevelopmental Disorders, University of Oslo and Oslo University Hospital, Oslo, Norway; 19 Melbourne Neuropsychiatry Centre, Department of Psychiatry, The University of Melbourne and Melbourne Health, Melbourne, Victoria Australia; 20 Department of Biomedical Engineering, The University of Melbourne, Melbourne, Victoria Australia; 21 Department of Psychiatry, University of Melbourne, Melbourne, Victoria Australia; 22 Department of Psychiatry, Bellvitge University Hospital, Bellvitge Biomedical Research Institute, Barcelona, Spain; 23 Centro de Investigación Biomédica en Red de Salud Mental, Carlos III Health Institute, Madrid, Spain; 24 Department of Social Psychology and Quantitative Psychology, Universitat de Barcelona, Barcelona, Spain; 25 Sant Pau Mental Health Research Group, Institut d’Investigació Biomèdica Sant Pau, Hospital de la Santa Creu i Sant Pau, Barcelona, Spain; 26 Department of Psychiatry and Forensic Medicine, Universitat Autònoma de Barcelona, Barcelona, Spain; 27 QIMR Berghofer Medical Research Institute, Brisbane, Queensland Australia; 28 Australian Characterisation Commons at Scale (ACCS) Project, Monash eResearch Centre, Melbourne, Victoria Australia; 29 Institute for Mental and Physical Health and Clinical Translation School of Medicine, Deakin University, Geelong, Victoria Australia; 30 Orygen, Melbourne, Victoria Australia; 31 Centre for Youth Mental Health, University of Melbourne, Melbourne, Victoria Australia; 32 Florey Institute for Neuroscience and Mental Health, Parkville, Victoria Australia; 33 Department of Neuroimaging, Centre of Neuroimaging Sciences, Institute of Psychiatry, King’s College London, London, UK

**Keywords:** Neuroscience, Psychology, Psychiatric disorders

## Abstract

The substantial individual heterogeneity that characterizes people with mental illness is often ignored by classical case–control research, which relies on group mean comparisons. Here we present a comprehensive, multiscale characterization of the heterogeneity of gray matter volume (GMV) differences in 1,294 cases diagnosed with one of six conditions (attention-deficit/hyperactivity disorder, autism spectrum disorder, bipolar disorder, depression, obsessive–compulsive disorder and schizophrenia) and 1,465 matched controls. Normative models indicated that person-specific deviations from population expectations for regional GMV were highly heterogeneous, affecting the same area in <7% of people with the same diagnosis. However, these deviations were embedded within common functional circuits and networks in up to 56% of cases. The salience–ventral attention system was implicated transdiagnostically, with other systems selectively involved in depression, bipolar disorder, schizophrenia and attention-deficit/hyperactivity disorder. Phenotypic differences between cases assigned the same diagnosis may thus arise from the heterogeneous localization of specific regional deviations, whereas phenotypic similarities may be attributable to the dysfunction of common functional circuits and networks.

## Abstract (web summary)

A new brain mapping approach tailored to individual people reveals that volume changes in psychiatric illness occur in highly variable locations across individuals, but that these differences often aggregate within common brain systems.

## Main

The neurobiological mechanisms of mental illness are elusive. Thousands of neuroimaging studies have documented diverse brain changes associated with specific psychiatric diagnoses, and meta-analyses have identified the brain regions that are most consistently affected by each condition, revealing both diagnosis-specific and transdiagnostic effects^1–7^. However, despite this substantial research effort, pathophysiological processes are poorly understood and clinically useful biomarkers are lacking.

One reason for this limited progress may be a continued reliance on case–control designs, which compare group averages and ignore the considerable clinical heterogeneity often shown by individuals with the same diagnosis^8,9^. Indeed, recent magnetic resonance imaging (MRI) studies investigating person-specific patterns of brain deviations show that group average differences are not representative of individual cases^10^. These individual-specific inferences are often performed using normative modeling^11,12^, which involves modeling normative expectations for a brain phenotype, such as gray matter volume (GMV), given an individual’s age, sex or other relevant characteristics. The model predictions can then be used to define a normative range of variation against which new individuals are compared. Fitting the model to data for multiple brain regions yields a personalized deviation map quantifying the extent to which each person deviates from population norms, thus enabling identification of areas associated with unusually small or large phenotypic values, termed extreme deviations, in an individual. Normative modeling studies of diverse MRI-derived phenotypes in attention-deficit/hyperactivity disorder^13^ (ADHD), bipolar disorder (BP), schizophrenia^10,14,15^ (SCZ) and autism spectrum disorder^16–18^ (ASD) have found that, while cases often have more extreme deviations than controls (HCs; defined as the absence of any clinical diagnosis), the specific location of these deviations varies considerably across individuals with the same diagnosis.

This extreme regional heterogeneity of individual brain deviations aligns with the well-described clinical heterogeneity often associated with specific psychiatric diagnoses^8,9^, but raises an important question: if cases show little overlap in the anatomical locations of their GMV deviations, what then explains phenotypic similarities between people assigned the same diagnostic label? It seems reasonable to assume that such similarities are driven by some common aspect of neural dysfunction across individuals, but the findings of normative modeling studies suggest otherwise.

One possible explanation is that these regionally heterogeneous deviations aggregate within common circuits or neural systems. The brain is a connected network, and pathological processes often affect distributed, interconnected systems^19–21^, meaning that it is possible for deviations in disparate loci to impact the function of common, connected areas. This principle has been demonstrated by lesion network mapping studies of neurological patients sharing a common motor, perceptual or cognitive syndrome^22,23^. Such patients typically show little overlap in the anatomical location of their lesions, but the lesioned sites are often functionally coupled to common areas (for a recent review, see ref. ^24^). The clinical expression of these syndromes is, therefore, more closely related to dysfunction of loci coupled to the damaged area, rather than dysfunction of the lesioned region itself.

**Fig. 1:**
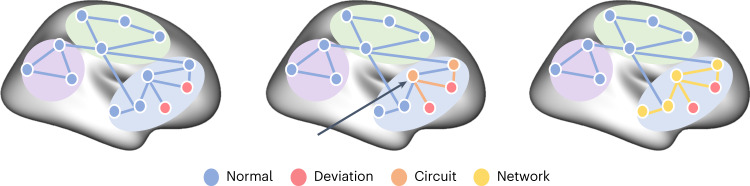
Characterizing neural heterogeneity at the level of brain regions, functional circuits and extended networks. A schematic showing how neural heterogeneity can be characterized at different scales. Nodes represent different brain regions, edges represent functional coupling (FC) between nodes and colored areas correspond to different functional networks of the brain. At the regional level (left), deviations from normative model predictions are localized to specific brain regions in each individual. Red nodes show the locations of such deviations mapped in two different people. A circuit-level analysis (middle) reveals areas that are functionally coupled to the deviant loci. In this work, we define a functional circuit as the set of regions that show significant FC with a specific deviant region (orange). In this example, the two deviant areas are coupled to a common region (black arrow) despite being located in different areas themselves. These circuits can be embedded within extended networks (right) that include regions that may not be directly coupled to the deviant regions, but which nonetheless participate within the same functional system (yellow).

In this Article, we considered whether a similar process is at play in psychiatric disorders by investigating whether anatomically heterogeneous regional brain deviations within psychiatric disorders are functionally coupled to common areas and networks. We developed a new framework to integrate normative models of GMV with elements of lesion network mapping to map the functional circuits and extended networks within which regional GMV deviations are embedded. We used this approach to derive a multiscale characterization of neural heterogeneity across 1,294 individuals diagnosed with one of six disorders: ADHD, ASD, BP, depression (MDD), obsessive–compulsive disorder (OCD) and SCZ. Inspired by studies of patients with brain lesions^22–24^, we tested the hypothesis that anatomically heterogeneous regional GMV deviations in each disorder are functionally coupled with common sites, either within a functional circuit or an extended functional network (for a schematic explanation, see Fig. 1). Our transdiagnostic, multiscale approach allowed us to comprehensively understand the extent of neural heterogeneity within each disorder while also revealing commonalities and differences between disorders.

## Results

### Sample characteristics

We examined data for 1,465 HCs (54.47% male) and 1,294 cases, taken from 14 different studies and 25 different scan sites. The clinical sample comprised 202 individuals with ASD (100% male), 153 individuals with ADHD (41.18% male), 228 individuals with BP (47.37% male), 161 individuals with MDD (34.16% male), 167 individuals with OCD (50.30% male) and 383 individuals with SCZ (62.14% male). The scanner details, sample size and demographic characteristics of each scan site, after various exclusions based on data quality and other criteria (Methods), are presented in Supplementary Table 1 (for age distributions, see Supplementary Fig. 1).

### Normative modeling

**Fig. 2:**
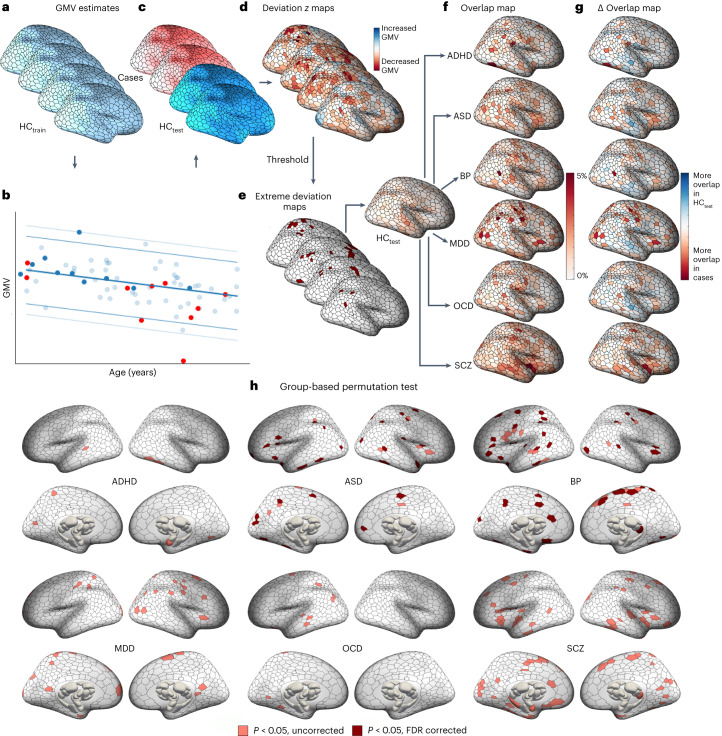
Regional heterogeneity of extreme negative GMV deviations in each disorder. **a**–**f**, Workflow for characterizing regional-level heterogeneity. GMV maps for each individual were parcellated into 1,000 cortical and 32 subcortical regions (**a**). The training dataset, HC_train_, was used to train a normative model to make predictions about regional GMV values given an individual’s age, sex and scan site (**b**). The predictions for held-out controls (HC_test_) and cases were then compared with empirical GMV estimates. Model predictions for one region, showing individuals in the training set (HC_train_; light blue) and the held-out control (HC_test_; dark blue) and clinical groups (red). Solid and dashed lines indicate the 99th and 95th centiles, respectively (**c**). For each individual, deviations from model predictions were quantified as a deviation $z$ map (**d**). This deviation map was then thresholded at $z > |2.6|$ to identify extreme deviations (**e**). For the HC_test_ and each clinical group, we quantified the proportion of individuals showing an extreme deviation in a given brain region, yielding an extreme deviation overlap map (**f**). We subtracted the HC_test_ overlap map from each clinical group’s overlap map to obtain an overlap difference map ($\Delta$ overlap map) for each clinical group and then evaluated the magnitude of this difference (for details, see Extended Data Fig. 3a–d) (**g**). Cortical and subcortical surface renderings showing regions with significantly greater overlap of extreme negative GMV deviations in cases compared with controls, as identified using group-based permutation tests (pink corresponds to ${P}_{{{\mathrm{uncorrected}}}} < 0.05$, red corresponds to ${P}_{{{\mathrm{FDR}}}} < 0.05$; two tailed, cases > controls) (**h**). Data used to generate this figure can be found in Supplementary Data 1 (Regional_neg_thr26).

We used an established pipeline to obtain voxel-wise estimates of GMV in each participant^25,26^ (Methods), which we aggregated into regional estimates for 1,032 brain areas^27,28^ (1,000 cortical and 32 subcortical regions; Fig. 2a). We then trained a normative model based on hierarchical Bayesian regression^29^ (HBR), fitted separately in each region, in a training set comprising 1,196 HCs (55.02% male; Fig. 2a; HC_train_) to establish a normative GMV range given an individual’s age, sex and scan site (Fig. 2b; for model fit statistics and site effects, see Supplementary Fig. 3 and Supplementary Table 2). HC_train_ spanned the age range of cases, enabling predictions for people aged between 18 and 64 years (Supplementary Fig. 2). The remaining 269 controls (52.04% male; age range 18–62 years) were held out as a test set (HC_test_) to establish a normative benchmark for comparison with each clinical group (Fig. 2c). For each individual in the clinical groups and HC_test_, we quantified the degree to which regional GMV estimates deviated from normative model predictions as a *z* score (termed Deviation *z* maps; Methods and Fig. 2d), with extreme deviations defined as $z > |2.6|$ (Fig. 2e), corresponding to $P < 0.005$, uncorrected, as per prior work^10^ (for a discussion of thresholding, see Methods).

**Extended Data Fig. 1:**
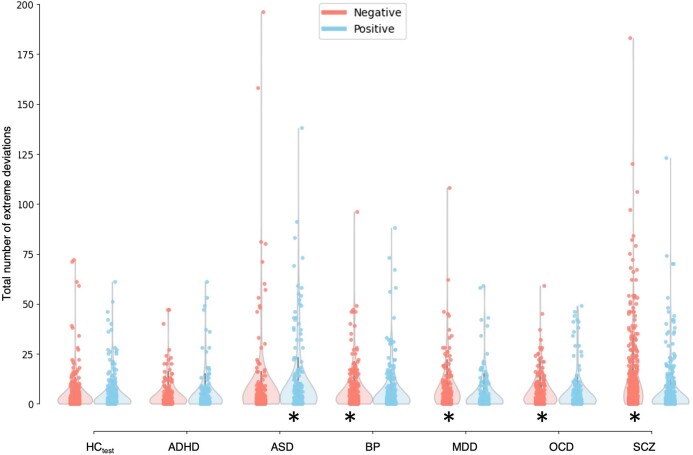
Distribution of person-specific positive (Z > 2.6; blue) and negative (Z < -2.6; red) deviation burden scores (that is, total number of extreme deviations) in each diagnostic group. * Indicates clinical groups showing a statistically significant difference in extreme deviation burden compared to the HC_test_ group (Mann Whitney U-test, *p* < .05, two-tailed).

Supplementary Table 3 shows that, across all groups, >75% of participants show at least one extreme negative deviation. The proportion of such participants was ~5–12% higher in MDD, SCZ, BP and OCD, compared with controls. Over 65% of participants showed at least one positive extreme deviation, and the proportion of such participants was ~4–10% higher in ADHD, ASD, BP and OCD compared with controls. People with BP, MDD, OCD and SCZ showed a higher burden of extreme negative deviations, defined as the total number of extreme deviations identified in each person, compared with HC_test_ ($P = 0.003$; Extended Data Fig. 1 and Supplementary Table 3). Only people with ASD showed a higher positive deviation burden than controls ($P < 0.001$; Extended Data Fig. 1 and Supplementary Table 3). Scan quality (for a definition, see Methods) was not correlated with extreme deviation burden (HC_test_: $\rho = 0.04,{P} = 0.51$; cases: $\rho = 0.03, {P} = 0.34$). In the following, we first focus on characterizing negative GMV deviations (that is, GMV values lower than normative expectations) before considering positive GMV deviations.

### Heterogeneity at the level of brain regions

**Extended Data Fig. 2:**
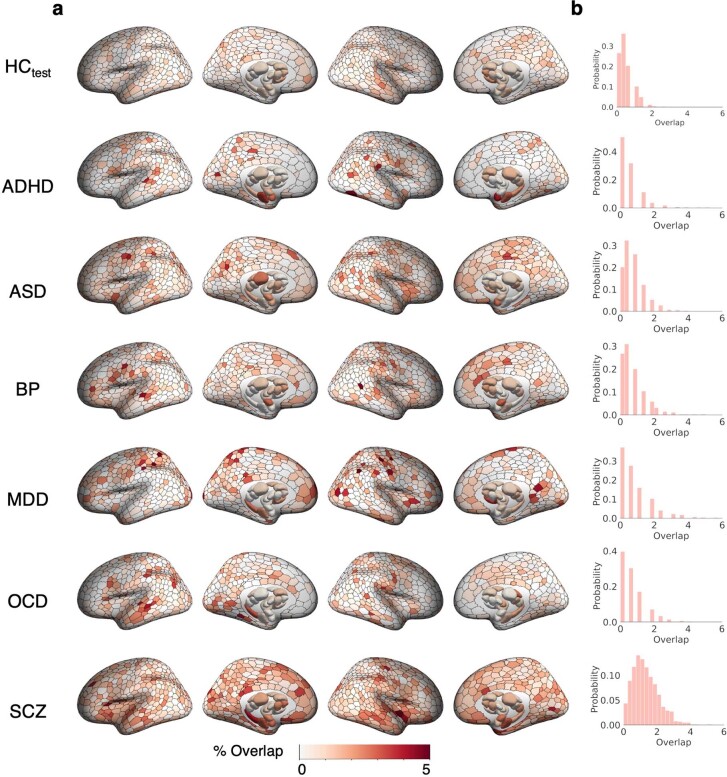
Spatial overlap of extreme negative GMV deviations (Z < - 2.6) in each group. a) Cortical and subcortical surface renderings showing spatial of overlap in 1032 brain regions, and b) the distribution of overlap percentages observed across all regions. Data used to generate this figure can be found in Supplementary Data 1*(Regional_neg_thr26)*.

We quantified regional heterogeneity in GMV deviations (Fig. 1, left) as the proportion of individuals showing an extreme deviation in each parcellated brain area, estimated separately for each diagnostic group and the HC_test_ cohort (Fig. 2f). Despite most people showing at least one deviation in each group, the maximum percentage overlap within any of the 1,032 brain regions never exceeded 7% (ADHD: 3.27%; ASD: 4.95%; BP: 5.26%; MDD: 6.21%; OCD: 4.19%; SCZ: 4.96%; HC: 2.97%). Hence, individual extreme deviations were common, but they were rarely found in consistent locations in individuals with the same diagnosis (Extended Data Fig. 2).

**Extended Data Fig. 3:**
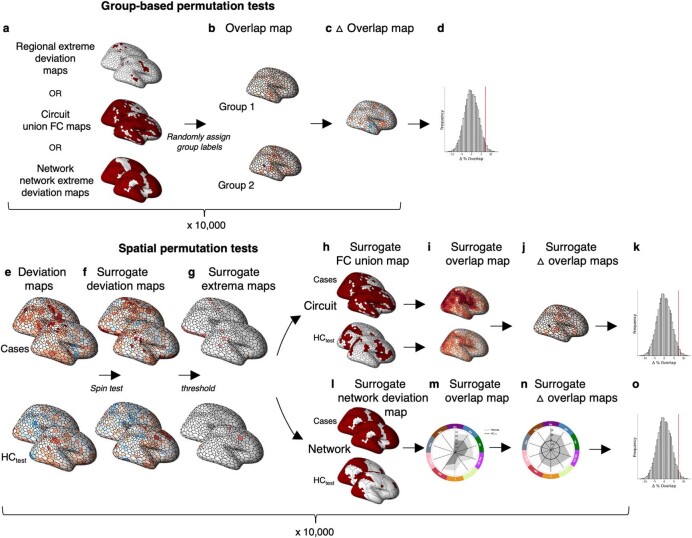
Permutation tests for evaluating region-level, circuit-level, and network-level overlap. We used two types of permutation test to evaluate different hypotheses. Group-based permutation tests were used to evaluate group differences in region-level, circuit-level, and network-level overlap, regardless of total deviation burden. These tests involved repeating each analysis 10,000 times after shuffling case and control labels. (a) At each iteration, we obtained a new grouping of person-specific deviation maps according to the shuffled group labels. At the regional level, we focused on extreme deviation maps (Fig. 2e); at the circuit-level, we focused on union FC maps (Fig. 3d); and at the network level we focused on extreme network deviation maps (Fig. 4c). (b) For each brain region, we computed an overlap map for the HC_test_ and each clinical group under shuffled group assignment (*Overlap map*). (c) We then subtracted the surrogate HC_test_ overlap map from the surrogate clinical group’s overlap map to obtain an overlap difference map *(Overlap map*). Steps (b) and (c) were repeated 10,000 times to derive an empirical distribution of overlap difference maps under the null hypothesis of random group assignment (d). For each brain region, we obtained *p*-values as the proportion of null values that exceeded the observed difference. The second type of permutation test we used was a spatial permutation test. (e) We used the unthresholded deviation maps of each person derived from the normative model to generate an ensemble of surrogate deviation maps for each individual in the test data (f). For cortical regions, the surrogate maps were generated using Hungarian spin tests^19,20^. For subcortical regions, we randomly shuffled deviation values across all subcortical areas (see Methods). (g) We then thresholded the null deviation maps (Z> |2.6|) to generate surrogate extreme deviation maps. To evaluate circuit-level group differences in overlap, (h) we obtained individual-specific surrogate FC union maps using the same procedure described in Fig. 3a-d. (i) For the HC_test_ group and each clinical group, we calculated surrogate within-group overlap maps. (j) We subtracted the HC_test_ surrogate FC overlap map from each clinical group’s surrogate FC overlap map to obtain a surrogate overlap difference map (*Overlap map*). Steps (f) – (j) were repeated 10,000 times to generate (k) a null distribution of circuit-level overlap difference maps for each disorder. To evaluate network-level group differences in overlap, (l) we obtained surrogate network-level extreme deviation maps using the same procedure described in Figure 5a-c. (m) For each clinical group and the control group, we quantified the proportion of individuals showing a surrogate deviation within each network (*Overlap map*). (n) We subtracted the HC_test_ surrogate network overlap map from each clinical group’s surrogate overlap (*Overlap map*). Steps (f) – (n) were repeated 10, 000 times to generate (o) a null distribution of overlap difference maps for each disorder. For all tests, statistically significance differences were identified using a threshold of *p*_*FDR*_ < 0.05, two-tailed.

We next compared the spatial overlap of each clinical group with controls by subtracting their respective percentage overlap values in each region (Fig. 2g). The statistical significance of the observed $\Delta$ overlap values in each region was assessed with respect to an empirical null distribution generated by shuffling group labels (Methods and Extended Data Fig. 3a–d). While each disorder showed isolated areas of greater overlap at uncorrected levels, only ASD (32 regions) and BP (45 regions) showed differences that survived false discovery rate (FDR) correction (${P}_{{{\mathrm{FDR}}}} < 0.05$, two tailed; Fig. 2h). These differences were scattered throughout the cortex and rarely aggregated into spatially structured clusters. Few regions showed significantly greater overlap in controls compared with cases (Supplementary Fig. 4). Repeating the analyses using a method that avoids reliance on a single threshold for defining extreme deviations^30^ yielded similar findings (Methods and Supplementary Figs. 5–7). Collectively, these results extend past reports^10,13,14^ to indicate that minimal spatial overlap in the location of person-specific extreme GMV deviations is a general characteristic of psychiatric illness.

### Heterogeneity at the level of functional circuits

**Fig. 3:**
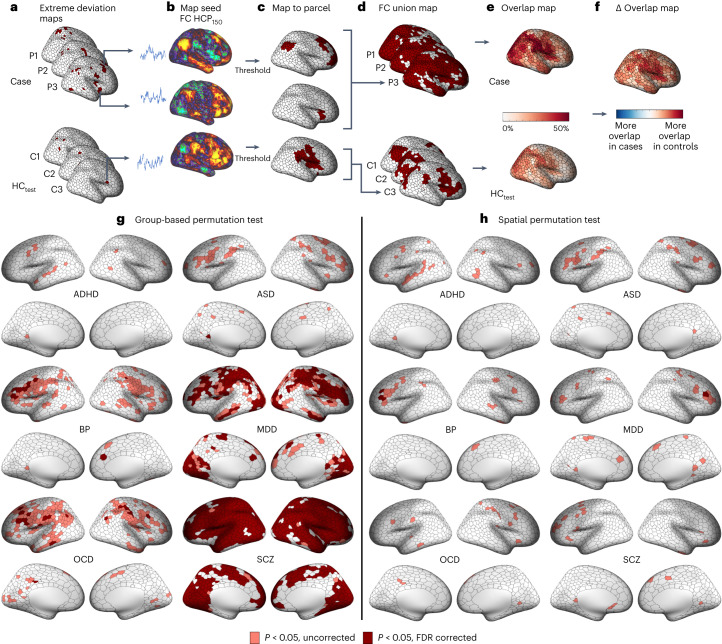
Functional circuit heterogeneity of extreme negative GMV deviations in each disorder. **a**–**f**, Workflow for characterizing circuit-level heterogeneity. For each participant in the HC_test_ and each clinical group, we took each brain region showing an extreme deviation (**a**). For each individual in an independent sample of controls (HC_150_), we extracted a representative time course from each deviant region and mapped the areas to which it is functionally coupled to using a seed-related FC analysis. Shown here are three participants in a clinical group (Case: P1, P2, P3) and three participants in the control group (HC_test_: C1, C2, C3). Two FC maps for two different deviant loci identified in P3, and one FC map for one deviant loci identified in C3 are depicted (**b**). We thresholded and binarized each FC map associated with a given extreme deviation (**c**). Note, that no subcortical regions survived this thresholding procedure. We took the union of the thresholded maps across all deviant FC maps to obtain a single map of all areas showing direct FC with one or more deviant areas for a given individual (**d**). For the HC_test_ and each clinical group, we quantified the proportion of individuals showing significant FC in a given region, yielding an extreme deviation FC overlap map (**e**). We subtracted the HC_test_ FC overlap map from each clinical group’s FC overlap map to obtain an FC $\Delta$ overlap map for each clinical group. Group differences in circuit-level overlap were evaluated with respect to two empirical null models (for details, see Extended Data Fig. 3) (**f**). **g**,**h**, Cortical surface renderings of regions with significantly greater overlap in cases compared with controls in areas functionally coupled to extreme deviations identified using group-based (**g**) or spatial permutation tests (**h**) (pink corresponds to ${P}_{{{\mathrm{uncorrected}}}} < 0.05$, red corresponds to ${P}_{{{\mathrm{FDR}}}} < 0.05$, two tailed, cases > controls). Data used to generate this figure can be found in Supplementary Data 1 (Circuit_neg_parc50).

**Extended Data Fig. 4:**
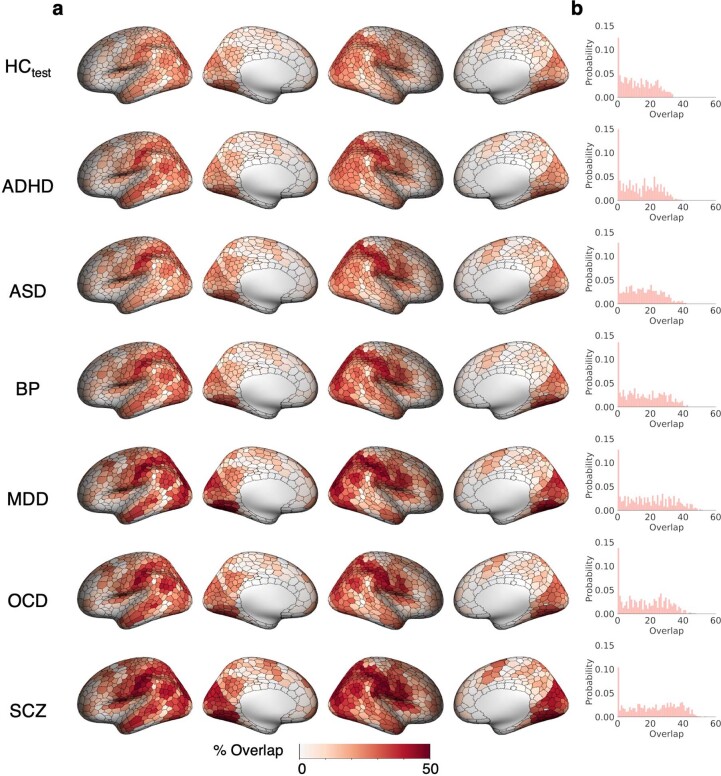
Spatial overlap of regions functionally coupled (vertex-wise threshold *p*_*FWE*_ < 0.025), to extreme negative deviations (Z < - 2.6) across groups, using a parcel-mapping threshold of 50%). a) Cortical surface renderings showing spatial overlap and b) the distribution of overlap percentages observed across all regions. Data used to generate this figure can be found in Supplementary Data 1*(Circuit_neg_parc50)*.

We next asked whether the regionally heterogeneous extreme deviations identified in each clinical group show significant functional coupling (FC) with common, remote areas, thus yielding greater interindividual consistency at the level of distinct functional circuits (Fig. 1, middle). To this end, we took each region showing an extreme deviation in each participant (Fig. 3a) and mapped its pattern of whole-brain FC in an independent sample of 150 unrelated HCs (HCP_150_) to establish the normative pattern of expected FC for the deviant region (Fig. 3b). We thresholded (${P}_{{{\mathrm{FWE}}}} < 0.025$) and binarized each deviant-related FC map (Fig. 3c), and took the union of these thresholded maps across all extreme deviations for a given person (Fig. 3d; for a discussion of thresholding issues, see Discussion), yielding a map that represents all areas showing significant FC with at least one extreme deviation in that individual. Next, we estimated, for each region, the proportion of individuals within each group for whom that region showed significant FC with an extreme deviation (Fig. 3e). This analysis revealed that the overlap observed in brain regions functionally coupled to deviant loci was much higher, in absolute terms, than the overlap observed in the locations of the extreme deviations themselves (Extended Data Fig. 4). For instance, the maximum circuit-level overlap observed across regions was 33% in HC_test_ and ranged between 39% (ADHD) and 53% (SCZ) in the clinical groups.

To a certain extent, one should expect overlap to be higher at the level of FC union maps (Fig. 3d,e) compared with regional deviation maps (Fig. 2e,f) since any single deviant area can show FC with multiple other regions, increasing the likelihood that common areas will be implicated across individuals. For this reason, regional circuit-level overlap maps in each clinical group must be contrasted with the HC_test_ overlap map, which provides a normative benchmark for the expected level of overlap in FC union values (Fig. 3f). A critical consideration in these contrasts concerns the effect of total deviation burden, which differs between groups (for example, Extended Data Fig. 1). For instance, the total number of extreme deviations identified in individuals diagnosed with SCZ was 4,951 compared with only 1,410 in HC_test_.

This discrepancy means that more deviant-related FC maps will be used in the FC union maps of SCZ individuals, thereby increasing the chance of observing higher overlap. On the one hand, this higher circuit-level overlap will have real phenotypic consequences, since a higher deviation burden is an intrinsic and expected characteristic of psychiatric disorders and these deviations are likely to impact circuit-level function and behavior. On the other hand, it is informative to determine whether the overlap is driven simply by group differences in deviation burden or reflects a preferential targeting of the circuit by the disorder in question. We therefore evaluated the statistical significance of regional group differences in circuit-level overlap using two different permutation tests designed to disentangle these effects (for details, see Methods and Extended Data Fig. 3). The first, group-based permutation test, relied on shuffling the group labels of the individual-specific FC union maps (Extended Data Fig. 3a–d) to assess differences in overall circuit-level overlap regardless of group differences in total deviation burden, thereby characterizing group differences in their ‘natural’ state. The second, spatial permutation test, evaluated group differences with respect to a null distribution that preserves the number of deviation-related FC maps contributed by each individual, thus matching differences in the total deviation burden of each group (Methods and Extended Data Fig. 3e–k). This analysis tests whether observed group differences in circuit-level overlap are greater than expected for FC maps generated from the same number of randomly chosen seeds, with the random seeds being selected from a deviation map with the same underlying spatial autocorrelation as the empirical deviation maps (for further details and interpretation, see Methods). In simple terms, the group-based permutation test identifies naturally occurring group differences in overlap regardless of deviation burden, whereas the spatial permutation test identifies differences in overlap beyond variations in overall deviation burden, implying a preferential targeting of specific functional circuits.

Using the group-based permutation tests, we observed significantly greater overlap across wide swathes of cortex for people with SCZ and MDD (${P}_{\mathrm{FDR}} < 0.05$, two tailed) compared with controls (Fig. 3g). Regions with significantly greater overlap in SCZ were distributed diffusely and included ~75% of cortical areas. In MDD, ~31% of areas were implicated, predominately localized to regions of visual, parietal, somatomotor, frontal and insula cortices. A similar spatial pattern was observed in ASD, OCD and BP when considering uncorrected results, although fewer regions survived FDR correction. Very few areas showed greater overlap of deviant-related FC in controls compared with cases (Supplementary Fig. 8a). An alternative method for mapping FC results to parcellated regions yielded comparable findings (Methods and Supplementary Figs. 9, 10a and 11a).

**Extended Data Fig. 5:**
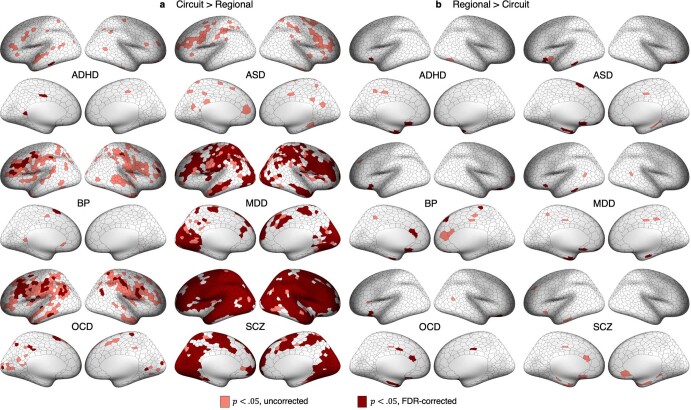
Comparison of regional and circuit-level heterogeneity. (a) Statistical maps showing regions where the case-control differences in overlap observed at the circuit level (that is, Fig. 3g) is significantly greater than the overlap difference observed at the regional level (for example, Fig. 2h), identified using the group-based permutation testing (pink corresponds to ${p}_{{uncorrected}} < .05$, red corresponds to ${p}_{{FDR}} < .05$, two-tailed, regional < circuit level overlap). (b) shows results for the reverse contrast (that is, regional > circuit level overlap). Data used to generate this figure can be found in Supplementary Data 1*(RegionalvCircuit)*.

Extended Data Fig. 5 confirms that, for nearly all areas where we identified greater circuit-level overlap in cases compared with controls (Fig. 3g), the magnitude of the difference exceeded the overlap differences observed at the regional level. This result aligns with our main findings to indicate that the level of overlap across participants was greater at the circuit compared with regional level, supporting the conclusion that GMV deviations in all psychiatric disorders except ADHD are part of common functional circuits, despite being located in anatomically heterogeneous areas.

We next used the spatial permutation test to determine the degree to which the above differences in circuit-level overlap were attributable to deviation burden. The results indicate that all disorders show some evidence of greater overlap in regions of left inferior and middle frontal gyri at uncorrected thresholds (Fig. 3h). However, only three areas in BP and one in MDD survived FDR correction. Few results survived FDR correction when considering regions showing greater overlap in controls (Supplementary Fig. 8b) and an alternative method for mapping FC results to our regional parcellation yielded comparable findings (Methods and Supplementary Figs. 10b and 11b). Thus, while these findings offer preliminary evidence for the preferential involvement of neural circuitry involving the lateral prefrontal cortex (PFC) in each disorder, the dominant factor explaining greater circuit-level overlap in cases is total deviation burden. In other words, cases show greater overlap at the circuit level largely because they are more likely to express extreme GMV deviations, which in turn increases the probability with which sites functionally coupled to deviant areas will be implicated.

### Heterogeneity at the level of extended functional networks

**Fig. 4:**
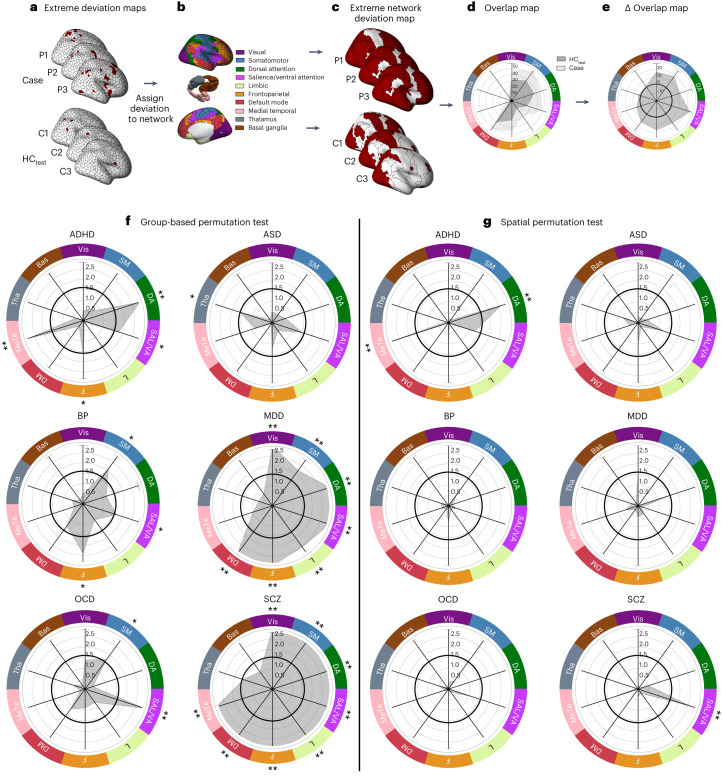
Functional network heterogeneity of extreme negative GMV deviations in each disorder. **a**–**d**, Workflow for characterizing network-level GMV heterogeneity. For each individual in the HC_test_ and each clinical group (**a**), we assigned each brain region showing an extreme deviation to one of seven canonical cortical functional networks or three subcortical nuclei (**b**), such that the entire network was considered deviant if it contained at least one region with an extreme deviation. The cortical surface renderings show the resulting network-level extreme deviation maps (**c**). We quantified the proportion of individuals in each group showing a deviation within each network and compared these proportions to the network overlap in HC_test_ (**d**). **e**, Group differences in network-level overlap were evaluated with respect to two empirical null models (for details, see Extended Data Fig. 3). **f**,**g**, The network-level −log_10_ *P* values associated with the difference in percent overlap for extreme negative GMV deviations between each clinical group and the HC_test_ cohort (gray) under group-based (f) or spatial permutation (g) testing, respectively. ** corresponds to ${P}_{{{\mathrm{FDR}}}} < 0.05$, two tailed, cases > controls, * corresponds to ${P}_{{{\mathrm{uncorrected}}}} < 0.05$, two tailed, cases > controls. The solid black line indicates −log_10_ *P* = 1.6 (*P* = 0.05, two tailed, uncorrected). VIS, visual; SM, somatomotor; DA, dorsal attention; SAL/VA, salience/ventral attention; L, limbic; F, frontoparietal; DM, default mode; MeTe, medial temporal; Tha, thalamus; Bas, basal ganglia). Data used to generate this figure can be found in Supplementary Data 1 (Network_neg_10network).

Our analysis thus far indicates that the locations of regional GMV deviations show marked individual heterogeneity, that cases show substantially greater overlap when considering the functional circuitry of these deviant loci and that this overlap is largely driven by total deviation burden. However, our circuit-level analysis only focused on areas showing significant FC with deviant regions, and these circuits often form part of larger, extended functional networks that may not be fully mapped by a circuit-level characterization (Fig. 1, right). We therefore examined the location of person-specific GMV deviations in relation to canonical functional networks, defined according to a widely used and validated classification of brain areas into one of seven cortical networks^27,31^ or three subcortical regions^28^, to derive a comprehensive, multiscale characterization of GMV heterogeneity (Fig. 4a–c). In this way, if an individual showed an extreme deviation in at least one region affiliated to a given network, the entire network was considered deviant (Fig. 4c). Once again, we quantified the proportion of individuals within each group showing a deviation in each network (Fig. 4d) and compared these proportions between the HC_test_ and each clinical group (Fig. 4e). We then evaluated group differences in network-level overlap using both group-based and spatial permutation tests, as was done in the circuit-level analysis (Extended Data Fig. 3).

The results of these analyses are shown in Fig. 4d–f (for a summary of the degree of spatial overlap (%) in each network for each group, see Supplementary Table 4). Using group-based permutation testing, SCZ and MDD cases showed significantly greater overlap in multiple networks compared with controls (${P}_{{{\mathrm{FDR}}}} < 0.05$, two tailed; Fig. 4f). For SCZ individuals, the differences included all networks except for the thalamus and basal ganglia. In MDD, all cortical networks were implicated. The dorsal attention network and medial temporal lobe were implicated in ADHD and the ventral attention network in OCD. No networks survived multiple comparison correction for ASD and BP. At uncorrected levels, the salience/ventral attention network was implicated across all disorders except for ASD. There were no networks in which controls showed greater overlap (Supplementary Fig. 12a).

Using spatial permutation tests with FDR correction, only the salience/ventral attention network showed greater overlap in SCZ, and the medial temporal lobe and dorsal attention networks showed greater overlap in ADHD (Fig. 4g). No deviant-related networks showed greater overlap in controls (Supplementary Fig. 12b).

We repeated the same analyses using a 20-network parcellation (17 cortical networks and 3 subcortical nuclei). The results of these analyses are shown in Supplementary Fig. 13 (also see Supplementary Table 5). Broadly, the findings align with those obtained using the ten-network parcellation to indicate that SCZ and MDD are associated with greater network overlap in negative GMV deviations across most networks, with the effects being more circumscribed in the other disorders. Using a 20-network parcellation, we found robust evidence for preferential involvement for temporoparietal and control networks in ADHD and MDD, respectively.

Together, these results align with the circuit-level analysis to indicate that SCZ and MDD are associated with greater network-level overlap of negative GMV deviations. Group differences in overlap are more circumscribed in ADHD, ASD, BP and OCD, although the salience/ventral attention network is implicated across most disorders. These effects are largely driven by total deviation burden, with only SCZ and ADHD showing robust evidence supporting preferential involvement of the salience/ventral attention and dorsal attention/medial temporal systems, respectively.

### Analysis of positive GMV deviations

**Extended Data Fig. 6:**
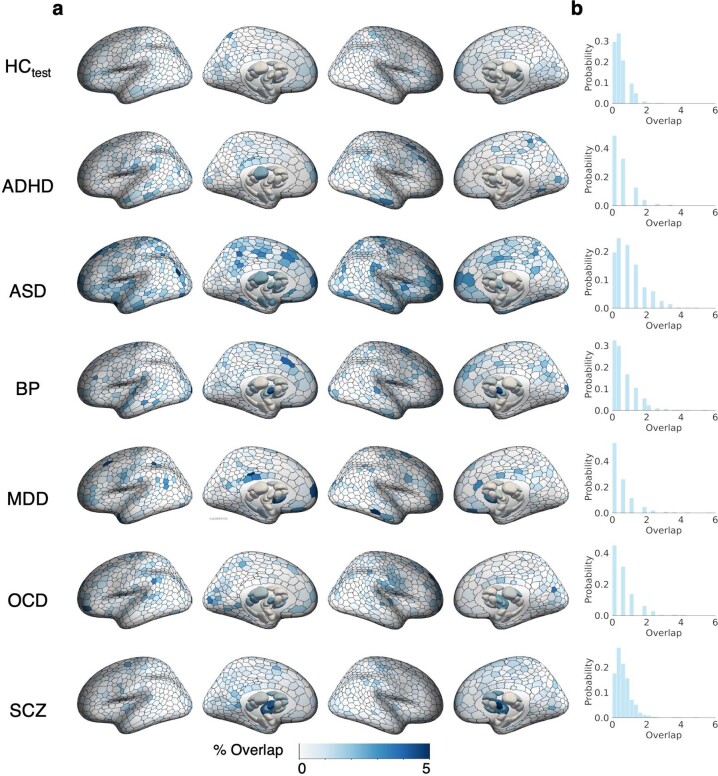
Spatial overlap of extreme positive GMV deviations (Z > 2.6) in each group. a) Cortical and subcortical surface renderings showing spatial of overlap in 1032 brain regions, and b) the distribution of overlap percentages observed across all regions. Data used to generate this figure can be found in Supplementary Data 1*(Regional_pos_thr26)*.

**Extended Data Fig. 7:**
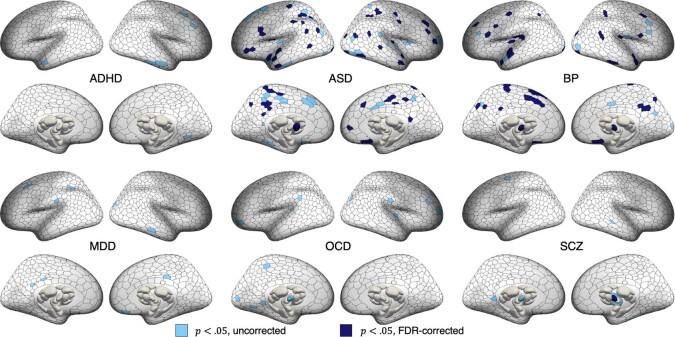
Regional heterogeneity of extreme positive GMV deviations in each disorder. Cortical and subcortical surface renderings showing regions with significantly greater overlap of extreme positive GMV deviations in cases compared to controls identified using group-based permutation tests (light blue corresponds to ${p}_{{uncorrected}} < .05$, dark blue corresponds to ${p}_{{FDR}} < .05$, two-tailed, cases>controls). Data used to generate this figure can be found in Supplementary Data 1*(Regional_pos_thr26)*.

For completeness, we repeated the same analyses as above for extreme positive GMV deviations, representing areas where volume was higher than normative expectations. At the regional level, extreme deviation overlap never exceeded 6% (ADHD: 5.23%; ASD: 3.96%; BP: 4.82%; MDD: 5.59%; OCD: 4.19%; SCZ: 5.22%; HC_test_: 2.60%) and very few regions showed significant case–control differences in overlap (Extended Data Figs. 6 and 7 and Supplementary Fig. 14).

**Extended Data Fig. 8:**
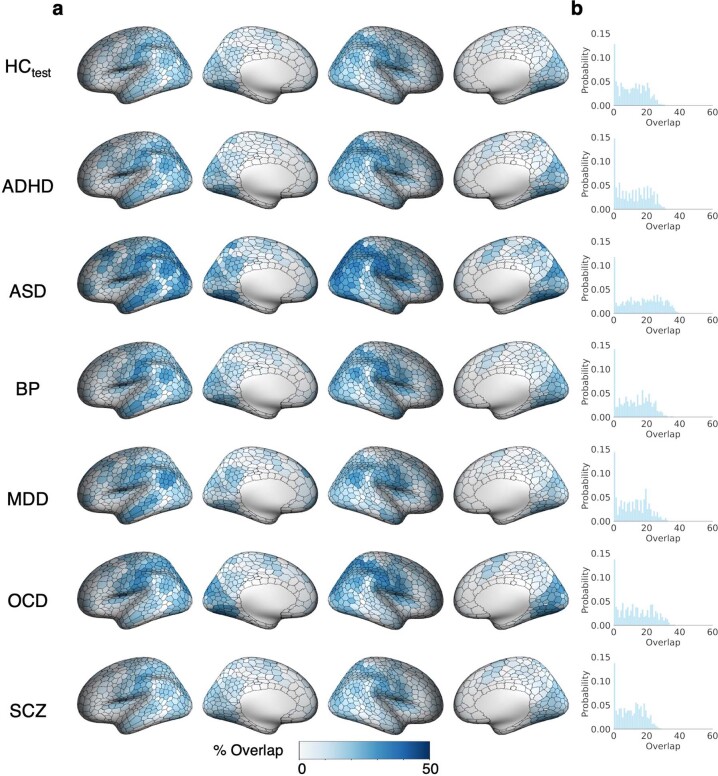
Spatial overlap in regions functionally coupled (vertex-wise threshold *p*_*FWE*_ < 0.025), to extreme positive deviations (Z > 2.6) across groups, using a parcel-mapping threshold of 50%). a) Cortical surface renderings showing spatial overlap, and b) the distribution of overlap percentages observed across all regions. Data used to generate this figure can be found in Supplementary Data 1*(Circuit_pos_parc50)*.

**Extended Data Fig. 9:**
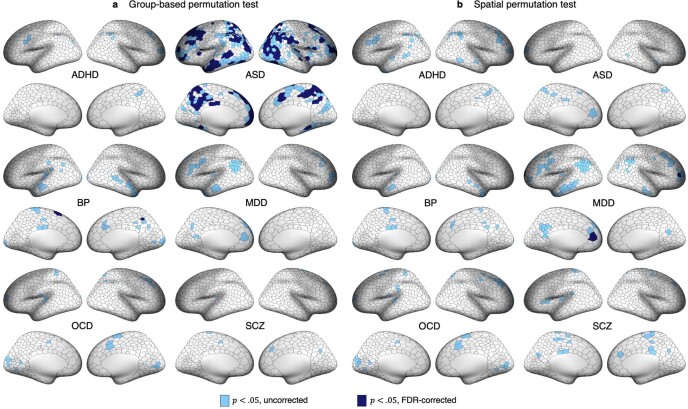
Regions showing greater overlap in areas functionally coupled to extreme positive GMV deviations in cases compared to controls. Group differences in circuit-level overlap were evaluated with respect to two empirical null models (see Extended Data Fig 3 for details). (a) and (b) show **c**ortical surface renderings of regions with significantly greater overlap in cases compared to controls in areas functionally coupled to extreme deviations identified using group-based or spatial permutation tests, respectively (light blue corresponds to ${p}_{{uncorrected}} < .05$, dark blue corresponds to ${p}_{{FDR}} < .05$, two-tailed, cases<controls). Data used to generate this figure can be found in Supplementary Data 1 (*Circuit_pos_parc50)*.

Circuit-level overlap was higher, reaching a maximum of 40% across all regions and disorders (Extended Data Fig. 8). Group-based permutation testing revealed significantly greater circuit-level overlap in individuals diagnosed with ASD compared with controls in ~20% of regions, predominately in visual, parietal and frontal cortices. No other differences survived FDR correction (Extended Data Fig. 9a and Supplementary Fig. 15a). Similarly, spatial permutation testing only identified isolated areas in pregenual cingulate and right lateral PFC as showing significantly greater overlap in MDD (Extended Data Fig. 9b and Supplementary Fig. 15b).

**Extended Data Fig. 10:**
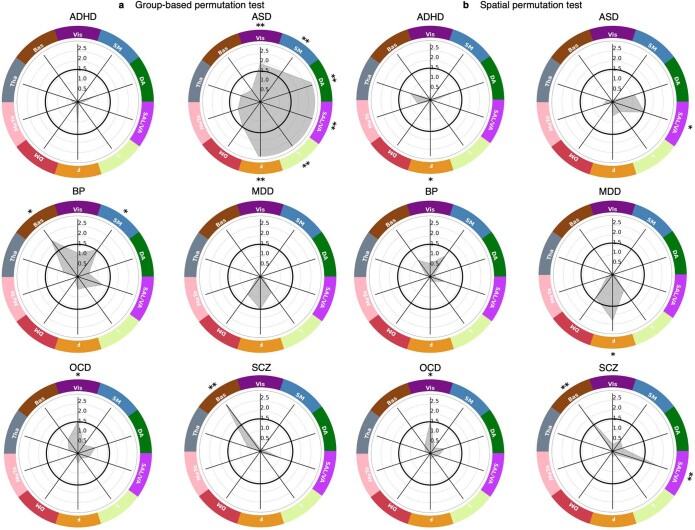
Functional networks showing greater overlap in extreme positive GMV deviations in cases compared to controls. The network-level -log_10_ p-values associated with difference in percent overlap for extreme positive GMV deviations between each clinical group and the HC_test_ cohort. ** corresponds to *p*_*FDR*_ < 0.05, two-tailed, cases>controls, * corresponds to *p*_*uncorrected*_ < 0.05, two-tailed, cases>controls. The solid black line indicates -log_10_ p = 1.6 (*p*=0.05, two-tailed, uncorrected). (a) and (b) identify networks showing significant differences under group-based or spatial permutation testing, respectively. Data used to generate this figure can be found in Supplementary Data 1*(Network_pos_10network)*.

At the network level, group overlaps were as high as 48% (Supplementary Table 5), with group-based permutation tests identifying significantly greater overlap in the basal ganglia in SCZ and all cortical networks except the default mode network in ASD ($P < 0.05$, two tailed), compared with controls (Extended Data Fig. 10a). Only the former difference was also observed with spatial permutation tests, and was also accompanied by greater overlap in the salience/ventral attention network (Extended Data Fig. 10b). The medial temporal lobe showed significantly greater overlap in controls compared with SCZ (Supplementary Fig. 16).

In summary, elevated circuit- and network-level overlap was particularly prominent in ASD under group-based permutation testing, and implicated areas of medial and lateral parietal, temporal and PFC at the circuit level, and all cortical systems except the default mode network at the network level. These differences were not apparent with spatial permutation testing, indicating that they were largely driven by the elevated positive GMV burden of individuals diagnosed with ASD. Differences in circuit- and network-level overlap for positive GMV deviations in other disorders were less pronounced.

## Discussion

We adapted lesion network mapping^22,23^ for use with normative modeling^11,12^ to characterize individual heterogeneity of brain deviations at the regional, circuit and network level in different psychiatric disorders. We showed that heterogeneity between individuals in regional GMV deviations is a general feature of psychiatric illness, but that these regionally heterogeneous loci are often embedded within common functional circuits and networks. Regional heterogeneity thus offers a plausible explanation for the well-described clinical heterogeneity observed in psychiatric disorders^8,9^, while circuit- and network-level aggregation of deviations is a putative neural substrate for phenotypic similarities between individuals assigned the same diagnosis. Using different null models, we showed that much of the elevated overlap observed at the circuit and network levels is attributable to total deviation burden. This result challenges prominent models in which specific disorders are assumed to arise from dysfunction of characteristic neural systems selectively targeted by the disease process^32^.

### Patient-specific deviations are regionally heterogeneous

Few areas showed significantly greater regional overlap in cases compared with controls, and no single area in any disorder showed an extreme deviation in more than 7% of cases across the 1,032 regions investigated. This substantial regional heterogeneity aligns with past normative modeling studies of GMV in SCZ, ADHD and BP^10,13,14^, confirming that this heterogeneity is a general feature of psychiatric illness. The variable involvement of different regions across individuals may yield distinct clinical profiles and drive phenotypic heterogeneity in people with the same diagnosis^8,9^. An important avenue of future work will involve precisely characterizing the relationship between GMV deviations and interindividual differences in symptom expression.

More generally, the high degree of regional heterogeneity observed indicates that group mean comparisons are not representative of the specific profile of GMV deviations apparent in any individual case. Mean comparisons may thus offer an incomplete account of causal pathophysiological mechanisms, unless the broader network context of any identified group differences is considered. Notably, most participants showed a low deviation burden (Supplementary Table 3). This result is expected, since the failure so far of psychiatric neuroimaging to identify robust diagnostic biomarkers of illness indicates that any disease-related brain changes are likely to be subtle and complex. We should, therefore, be circumspect about our ability to identify strong neurobiological signatures of psychiatric disorders.

### Deviations aggregate in common circuits and networks

Despite considerable heterogeneity at the regional level, deviations were often coupled to common functional circuits and networks. In some cases, >50% of people with the same diagnosis showed a deviation implicating at least one of these systems, with group-based permutation testing indicating that all disorders showed some evidence of greater circuit-level overlap than controls. This higher overlap at circuit and network levels parallels lesion network mapping studies of neurological syndromes, which suggest that many clinical phenotypes are not caused by dysfunction of the lesioned area itself but by its impact on remote, functionally coupled regions^22–24^. Our findings indicate that a similar process may occur in psychiatric disorders, with anatomically distributed GMV deviations often being coupled within similar circuits and networks. The consequent dysfunction of these common circuits and networks may drive clinical similarities between people with the same diagnostic label, despite extreme heterogeneity in the locations of the deviations themselves. Multiple mechanisms may explain these circuit- and network-level effects, ranging from transient diaschisis-like effects on distributed circuit/network function through to more prolonged transneuronal dysfunction caused by aberrant interregional signaling or disrupted axonal transport of trophic factors^20^.

Areas of frontal, parietal, insula and temporal cortex showed greater circuit-level overlap across most conditions, and cross-disorder differences were more of degree rather than kind. For instance, differences in circuit-level overlap were spatially circumscribed in ADHD but encompassed nearly the entire brain in SCZ (Fig. 3g). These findings challenge classical views that distinct psychiatric diagnoses are associated with dysfunction in specific circuits (for example, ref. ^32^,) and suggest that each disorder is associated with complex changes that affect diverse neural systems^33,34^, often transdiagnostically^35–37^. Accordingly, our network-level analysis revealed greater overlap in the salience/ventral attention network in five of the six disorders that we considered (Fig. 4f). The salience/ventral attention network plays a central role in cognitive control^38^, interoceptive awareness^39^, and switching between internally and externally-focused attention^40^. Its dysfunction has been implicated in a diverse range of psychiatric disorders^41–44^, is associated with increased levels of general psychopathology in youth^45^ and shows cross-disorder abnormalities in meta-analyses of classical case–control voxel-based morphometry (VBM) studies and functional neuroimaging studies^35–37^. Our findings support this past work to suggest that salience/ventral attention network dysfunction may play a critical role in the expression of general psychopathological processes common to diverse diagnoses^46^.

### Generic and preferential targeting of neural systems

Our spatial permutation test allowed us to evaluate the degree to which circuit- and network-level overlap was explained by group differences in total deviation burden. At the circuit level, prefrontal regions were identified as showing greater overlap compared with controls in all clinical groups at uncorrected levels, consistent with extensive evidence for prefrontal dysfunction in each condition^47–49^. However, only regions of right and left lateral PFC in MDD and BP, respectively, survived FDR correction. The right PFC showed 21% overlap in MDD cases, whereas the left PFC showed 14–15% overlap in BP. Extensive literature implicates lateral PFC dysfunction in affective disorders^48,50,51^, and the dorsolateral PFC is a popular brain stimulation target in both MDD^52^ and BP^53^. The region has also been implicated by lesion network mapping as a core site explaining the emergence of depression following stroke^54^. Given that our spatial permutation test did not find any evidence of greater network-level overlap in MDD and BP, our findings point to a strong specificity for functional circuits coupled to these particular prefrontal areas. Spatial permutation testing also revealed greater network-level overlap for SCZ in the salience/ventral attention network and for ADHD in the dorsal attention network and medial temporal regions, suggesting that GMV deviations preferentially target these systems in a disorder-specific way.

Lesion network mapping of neurological cases indicates that symptom expression is often driven by dysfunction of remote sites connected to a lesion, implying that these remote sites represent viable treatment targets^22,23,55,56^. Following this logic, the findings from our spatial permutation analysis imply that viable treatment targets may be located in the right PFC for MDD, in the left PFC for BP, in the salience/ventral attention network for SCZ, and in the dorsal attention and medial temporal networks for ADHD. However, these targets may only be relevant for a subset of patients, with the observed overlaps ranging between 10% and 50% across disorders. It thus follows that the most current approaches, which attempt to identify a single common therapeutic target for each diagnosis, will only have limited success. A more comprehensive understanding of patient-specific brain changes, and their network context, will be necessary to develop more effective, and personally tailored, interventions.

In most other cases, we failed to reject the spatial null hypothesis. This result indicates that many case–control differences in circuit/network overlap observed under group-based permutation cannot be attributed to the preferential accumulation of deviations within a particular circuit/network, since the differences are consistent with comparisons of the same number of randomly selected seeds in each group. When taken with our group-based permutation analysis, our findings indicate that accumulating deviations are more likely to be coupled to areas in prefrontal, temporal, parietal and insula cortices, simply because these areas are known connectivity hubs of the brain and have a higher probability of being functionally coupled to other regions^57–59^. While this circuit- and network-level accumulation will still have real phenotypic consequences, the spatial permutation test allowed us to identify an underlying random process as a candidate generative mechanism. This finding challenges the implicit assumption in many studies that any brain changes observed in a disorder result from a targeted pathophysiological process. Further work investigating the genetic and environmental contributions to individual-specific deviations should elucidate the mechanisms driving their anatomical distribution.

### Heterogeneity of positive deviations

The locations of positive GMV deviations were more heterogeneous than negative deviations, and showed less overlap across people. The exceptions were ASD and BP at the regional level, which showed significantly greater overlap in predominately frontal, parietal areas. At the circuit level, ASD showed significantly greater overlap in visual, parietal and frontal cortices, and at the network level, all cortical networks except the default mode network were implicated in ASD. ASD has been associated with dysregulated and accelerated brain growth, particularly in the temporal, parietal and frontal association cortices during early childhood^60^. Whether these increases persist into adulthood and can explain the present findings remains unclear.

Differences in circuit-level and network-level overlap for positive GMV deviations in other disorders were less consistent. In MDD, greater circuit-level overlap in left pregenual cingulate and anterior right lateral PFC is consistent with the known roles of these areas in regulating emotion^61^ and cognitive control^62^, respectively. The greater overlap observed in the basal ganglia of people with SCZ may be attributable to the effects of antipsychotics, which can cause volumetric expansion in this region^63^.

### Limitations

While deviation burden was higher in cases than controls, and most people showed at least one deviation, a substantial fraction of clinical participants (~50%) showed a relatively low deviation burden (<3 deviations). This result implies that there is considerable overlap between cases and controls, as noted for many other phenotypes^64^, and that brain changes associated with psychiatric illness may be subtle and complex. This subtlety, combined with considerable interindividual heterogeneity, may partly explain the failure of the field to identify pathognomonic biomarkers for psychiatry, despite decades of research. The result may also reflect the limited sensitivity of MRI-derived GMV estimates to map pathophysiologically relevant brain changes. Normative modeling of other phenotypes, such as those obtained with functional or molecular imaging, may reveal stronger separation between groups.

Sample sizes in single-site psychiatric neuroimaging studies are often small, so we pooled data from multiple sites to generate a sufficiently large cross-disorder dataset. As a result, the data were collected with different acquisition, recruitment and clinical assessment protocols. To avoid introducing a dependence between scan site and diagnostic group, which can confound case–control comparisons, we focused primarily on control data obtained on the same scanners as the clinical data. While our model diagnostics indicated adequate fit to the data, future work could extend the techniques used here so that they can be used with normative models trained on larger samples (for example, refs. ^65,66^) to obtain more reliable deviation estimates.

Although we used stringent quality control, and our hierarchical Bayesian model^29^ appropriately parsed site-related variance (Supplementary Table 2), investigating correlations with symptom profiles or other clinically relevant variables such as age of onset, illness duration, medication exposure or disease severity across disorders was beyond our scope. Indeed, while our methods are useful for characterizing circuit- and network-level overlap across individuals, they are not well suited for analyses of individual differences in behavior. A valuable extension of our work would involve directly testing the hypothesis that circuit- and network-level overlap relate to clinical similarities between patients. Addressing this goal would require appropriate methods for aggregating deviation-related circuit- and network-level data within and between individuals, and extensive measurement of diverse symptom dimensions to allow adequate assessment of clinical phenotypes^67^. Extensive symptom sampling that allows for personalized models would be particularly useful in this context^68^. Such data are rarely available in single-site clinical neuroimaging studies. Future studies may work toward developing harmonized, transdiagnostic and multisite clinical protocols that can be used across different disorders, such as those informed by the Hierarchical Taxonomy of Psychopathology^69^. The specific age at which the measures are acquired should also be considered, given that the nature and magnitude of case–control differences may vary across the lifespan^4^. Such an approach may facilitate data-driven strategies to identify biological subtypes based on patient-specific deviation maps that cut across traditional diagnostic boundaries.

Our cases were diagnosed according to Diagnostic Statistical Manual of Mental Disorders (DSM) or the International Classification of Diseases (ICD) criteria^70,71^. Given the widespread application of DSM and ICD in clinical and research contexts, we deemed it important to understand heterogeneity with respect to these constructs and to gain insight into the neural correlates of phenotypic similarities and differences between cases assigned specific diagnoses. Nonetheless, focusing on specific syndromes rather than traditional diagnoses, as in the lesion network mapping literature^23,24^, may yield a more precise mapping between regional deviations, their network context and behavior. Establishing sufficiently large databases for conducting such syndrome-focused analyses will be a key challenge for future work.

### Conclusions

Our multiscale analysis of neural heterogeneity across six psychiatric disorders confirms that extreme regional heterogeneity of GMV deviations is a general characteristic of mental illness. Further, we showed that these deviations are often coupled to common functional circuits and networks, offering a putative neural substrate for phenotypic similarities among individuals assigned the same diagnosis. The common involvement of prefrontal and parietal circuits, and the salience/ventral attention network, across disorders may be a marker of transdiagnostic psychological distress, with the variable involvement of other systems explaining phenotypic differences among disorders. More broadly, our findings underscore the need to consider the network context of disorder-related markers of pathophysiology^20^, and indicate that the clinical expression of disease is not driven solely by sites of primary pathology, but also by the effect of this pathology on remote, connected systems.

## Methods

Original study protocols were approved by the local ethics committee of each dataset, and written informed consent was obtained from each participant. This current study was approved by the Monash University Research Ethics Committee (project ID: 23534). In this study, data collection and analysis were not blind to the conditions of the experiment because participants were not placed into experimental groups.

### Participants

This study included 3,746 individuals (1,865 HCs; 1,833 cases across six different diagnostic categories) from 14 separate, independently acquired datasets and 25 scan sites. No statistical methods were used to predetermine sample sizes but to our knowledge, our dataset is one of the largest aggregations of case–control data outside the ENIGMA consortium^111^. Full details on the study design and clinical characteristics have been described previously for each dataset (for basic characteristics and relevant references, see Supplementary Table 1). Each study was approved by the relevant ethics committee and written informed consent was obtained from each participant. Participants were reimbursed for their time in accordance with the local research protocols and ethics committee guidelines.

The final sample was drawn from a larger pool of individuals recruited across the 14 datasets. In addition to the specific quality control procedures used within each study (references in Table 1), we performed a series of quality control checks and exclusions for our analysis. Specifically, we excluded participants if they were below 18 years or above 64 years of age (*N* = 346); did not have the necessary clinical data (clinical diagnosis or, for HCs, absence of any clinical diagnosis) or demographic information (age, sex and scanner site, *N* = 53); if their T1-weighted structural MRI scan did not survive our stringent manual and automated quality control procedure, as explained below (*N* = 269); or if the data came from a site with less than ten individuals in the same group and sex (described in the Normative model section, below; *N* = 217). Our final sample available for analysis thus comprised 1,465 HCs and 1,294 cases. Demographic and other details of this cohort are presented in Supplementary Table 1.

## Mapping neural heterogeneity at the regional level

Our analysis aimed to characterize neural heterogeneity at the level of individual brain regions, neural circuits and extended brain networks (Fig. 1). To map heterogeneity at the regional level, we obtained person-specific deviation maps, representing the extent to which the GMV of a given person within each brain region deviates from normative predictions. This analysis involved quantifying regional GMV for each individual and evaluating the observed measures with respect to an underlying normative model, as outlined in the following.

### Anatomical data

We estimated regional GMV using VBM of T1-weighted anatomical MRI scans^25,26^. We focused on GMV because it is one of the most frequently studied neural phenotypes in psychiatry. VBM is also arguably the most widely used tool for measuring GMV in this context. Image acquisition parameters for each dataset are provided in Supplementary Table 1. The following outlines our quality assurance procedures and data processing pipeline, which was applied to all raw T1-weighted images obtained for each dataset.

### Quality control

All T1-weighted images were visually inspected and evaluated for the presence of artifacts^72,73^, resulting in the exclusion of 53 images with gross artifacts or abnormalities. Next, we used the Computational Anatomy Toolbox^25^ (CAT12 r1113, ref. ^74^) to generate a weighted overall image quality rating (IQR) for every scan. This metric combines ratings of basic image properties, including the level of noise and geometric distortions, into a single score that quantifies the overall image quality of a participant’s T1-weighted scan (for more information, see ref. ^74^). On this metric, lower scores denote higher image quality. As per previous work^10^, we excluded 153 images with an IQR >2.8. An additional 63 images were excluded due to a failure of our processing pipeline. Collectively, this quality assurance process resulted in the exclusion of a total of 269 scans

### MRI preprocessing

Regional GMV was estimated using the CAT12 VBM pipeline^25,26^, which is included as an extension of Statistical Parametric Mapping software v7771^75^ in MATLAB v9.8 (ref. ^76^). Briefly, the T1-weighted images were first corrected for intensity nonuniformities, and segmented into gray matter (GM), white matter and cerebrospinal fluid tissue probability maps. Then, using the high-dimensional Diffeomorphic Anatomical Registration Exponentiated Lie Algebra^77^, the segmented scans were normalized into standard Montreal Neurological Institute IXI555 space. Lastly, the images were bias-field corrected and modulated by the linear and nonlinear components of the Jacobian determinant obtained from the Diffeomorphic Anatomical Registration Exponentiated Lie Algebra deformation fields to obtain voxel-wise estimates of GMV. To constrain our analyses to GM voxels, we generated a mean image from all the normalized GM maps and retained voxels with a tissue probability ≥0.2.

### Brain parcellation

We employed HBR to estimate normative models of regional GMV (see the Normative Model section, below). To limit computational burden, we parcellated the brain into 1,032 cortical and subcortical regions by combining well-validated parcellations of the cortex^27^ comprising 1,000 regions, and of the subcortex^28^ comprising 32 regions. The cortical parcels have been mapped to well-described, canonical functional networks of the brain^31^, facilitating our analysis of network-level overlap in deviations.

Recent work indicates that group-based parcellations can be tailored to capture individual variability in regional borders (for example, ref. ^78^). We used a group-based parcellation to ensure a consistent approach across cortex and subcortex (methods for individual subcortical parcellations have not yet been developed), to allow direct comparison with extant literature (which have overwhelmingly relied on group parcellations), and to ensure that region-of-interest seeds remained approximately the same size among participants, as the effect of varying seed sizes between individuals has not yet been studied extensively. Investigating the effect of individually tailored parcellations on our findings will be an important goal for future work. Voxels that overlapped between atlases were assigned to the corresponding subcortical region. Regional GMV estimates were obtained using freely available code^79^.

The choice of parcellation will necessarily affect the degree of overlap observeable in any given brain area, such that coarser parcellations will be associated with higher levels of overlap. Comparison with the control data thus provides a critical normative benchmark against which to evaluate the levels of overlap observed in each clinical group. We chose our 1,032-region parcellation to offer high spatial resolution while ensuring computational feasibility. The same parcellation was used in regional-, circuit- and network-level analyses, facilitating direct comparison across scales.

### Normative modeling

We used normative modeling to obtain person-specific GMV deviation maps in relation to an underlying model of normative expectations for regional GMV variations (see refs. ^11,12,29^, PCNtoolkit v0.16 (ref. ^80^)). Normative models estimate the mean and variance (referred to as a normative range) of a response variable (for example, GMV) from a set of clinically relevant covariates (for example, age and sex) across a large healthy sample, referred to as a training set. These estimates are then used to quantify the deviations of samples in the test subset, which typically consists of cases sampled from the normative demographic range of the training set.

When using multisite data, scanner- and site-related variability introduce artefactual variance that confounds the results of any subsequent analyses^81^. These confounding effects present as site-correlated biases that cannot be explained by biological heterogeneity between samples. To account for these site-related effects, we built our normative model using HBR, which successfully accommodates signal and noise variance in multisite data by estimating different but connected mean and variance components through shared prior distributions across sites^29^. Before modeling, regional GMV estimates were first subjected to a Box–Cox transformation to ensure normality^82^. The optimal lambda parameter for minimizing skewness was estimated for each brain region independently using maximum likelihood.

For the model to accurately parse variance attributable to age, sex and site, sufficient observations are required for any given combination of these variables. We therefore excluded data cells containing less than ten HCs for a particular sex at a given site. If this exclusion procedure resulted in less than ten cases in total from any given site, the entire site was excluded, resulting in the exclusion of 217 scans.

The training data for the normative model (HC_train_) were created by randomly selecting 90% of HC individuals from each site, provided that the site included data for ≥30 HCs. For sites with smaller HC samples, all HC data were included in the training set. The test data (HC_test_), which was completely independent of the normative model, comprised the 10% of HCs from sites with ≥30 HCs and all case scans. The HC_test_ data, comprising 269 individuals (140 male), offered a normative benchmark for assessing case-specific model deviations, as outlined below.

Following stratification of our sample into training and test subsets, for each of the 1,032 parcellated brain regions, we fitted separate HBRs^29^. The HBRs modelled site and sex effects with random slopes, intercepts and noise, to model GMV as a function of age, sex and site in the training data, yielding estimates of normative regional GMV variance and predictive uncertainty. Importantly, based on a partial-pooling approach, shared prior distributions were imposed over site-specific and sex-specific model parameters. These shared priors assume that, while the model parameters for each site and sex are different, they are drawn from a common distribution. These shared priors thus regularize the model parameters and prevent the model from overfitting small batches for a given sex or site.

To quantify deviations from the normative model predictions for each participant in the test data, we generated deviation *z* maps for each participant. Specifically, for each participant *i*, at each brain region $j$, we combined the predicted GMV $\widehat{{y}_{ij}}$, true GMV ${y}_{{ij}}$, the predictive uncertainty ${\sigma }_{{ij}}$ and the normative variance ${\sigma }_{{\rm{nj}}}$ to calculate a *z* score, ${z}_{{ij}}$, as

$${z}_{{ij}}=\frac{{y}_{{ij}} - \widehat{{y}_{ij}}}{\sqrt{{\sigma }_{{ij}}^{2} + {\sigma }_{{nj}}^{2}}},$$

which quantifies the extent to which an individual’s regional GMV estimate deviates from the model prediction, given the uncertainty of the model.

For any given brain region, we are interested in participant values that show large deviations from the normative expectations set by the model, under the assumption that these deviations represent pathophysiologically relevant features of the disease^11^. We used two complementary approaches to characterize large positive and negative deviations from the normative model at each region and for each individual. First, we thresholded the deviation maps at $z > |2.6|$ (that is, $P < 0.005$) to identify regions showing extreme deviations from model predictions. These deviation maps were derived for each individual in the clinical and the held-out control (HC_test_) groups and were used for subsequent analyses. We used this approach to be consistent with prior work^10,14^ and because alternative methods, such as the FDR^83^, rely on adaptive thresholds that can vary between individuals. Applying a fixed *z* threshold allows for an absolute definition of extreme deviations. Second, to ensure that our findings were not driven by this specific choice of threshold, we adapted a threshold-weighted approach^30^, as detailed below.

Note that scaling deviation $z$ scores by predictive uncertainty means that we are less likely to observe deviations in data regimes where model uncertainty is high. This may be a consideration for deviation estimates in older participants, given that our training sample was skewed toward younger ages. However, we found no correlation between age and deviation burden ($\rho = -0.004,{P} = 0.86$).

### Evaluation of model performance

To assess model generalizability, we used five-fold cross-validation applied to the HC_train_ cohort (*n* = 1,196 HC individuals, 55% male). Specifically, we partitioned the HC_train_ cohort into five folds. Within each fold, we trained HBR models on 80% of participants, using age, sex and site as covariates, withholding 20% of the participants for estimating generalization performance. This procedure was repeated five times so that regional GMV values for all participants in the HC_train_ group were predicted once. As above, we estimated deviation *z* maps for every individual and identified extreme deviations. This procedure is standard in machine learning and provides approximately unbiased estimates of the true generalization ability.

For each fold in the cross-validation, we assessed model fit for each brain region by evaluating three performance metrics: (1) explained variance, (2) the mean standardized log-loss and (3) the standardized mean-squared error^29^, as shown in Supplementary Fig. 3. We also evaluated the model’s efficacy in partitioning site-related variance in the data using linear support vector machines (LSVMs). Specifically, we used a series of one-versus-all LSVMs (with default slack parameter of 1) trained separately on the *z* maps from the HC_train_ and the HC_test_ subsets to classify scan sites. For each site, we ran a two-fold LSVM classifier to obtain the mean balanced accuracy score for the given site. Here, a balanced accuracy at chance level (50%) indicated that the resulting deviations were not contaminated by residual site effects, as confirmed in Supplementary Table 2. To assess whether individual variations in scan quality affected the deviation *z* maps, we calculated the Pearson’s correlation between the total number of extrema and CAT12’s IQR rating in each group.

### Characterizing regional heterogeneity of extreme deviations

We characterized the heterogeneity of case-specific thresholded deviation maps for each disorder using a nonparametric approach. Specifically, for each clinical group and the HC_test_ cohort, we computed the proportion of individuals in each group showing an extreme deviation within each region, resulting in group-specific overlap maps estimated separately for positive and negative extrema (Fig. 2f). We used the proportion of individuals rather than raw counts to account for sample size differences between groups. Next, we subtracted the HC_test_ overlap map from each disorder’s overlap map, resulting in an overlap difference map for each disorder (Fig. 2g). We then permuted group labels (that is, HC and case) and repeated the procedure 10,000 times to derive an empirical distribution of overlap difference maps under the null hypothesis of random group assignment (Extended Data Fig. 3a–c). For each brain region, we obtained *P* values as the proportion of null values that exceeded the observed difference (Extended Data Fig. 3d). The tails of the null distribution (that is, values associated with $P < 0.10$) were approximated using a generalized Pareto distribution^84^, as implemented in the Permutation Analysis of Linear Models software package^85^ (alpha116), to allow inference at arbitrarily high levels of precision. Statistically significant effects were identified using an FDR-corrected^83^ threshold of ${P}_{{{\mathrm{FDR}}}} < 0.05$, two tailed.

### Threshold-weighted deviation mapping

The procedure to characterize the regional heterogeneity of extreme deviations above used an arbitrary threshold ($z < |2.6|$) on each individual *z* map. The practical benefit of this approach is that it allows calculation of intuitive metrics, such as the proportion of cases within a group showing a supra-threshold deviation within a given brain region. We complemented this analysis with an alternative approach, which does not yield similarly intuitive metrics but which integrates results across a range of thresholds^30^, thus allowing us to determine the degree to which our findings depend on a specific threshold choice. First, for each diagnostic group, we thresholded *z* maps across the threshold range $1.64 < z < 3.10$, in 100 equal log-space increments, separately for positive and negative extrema. Second, we obtained a region- and threshold-specific percentage overlap map quantifying the proportion of individuals within each group showing an extreme deviation for any given region and threshold. Third, we applied a weighted function to penalize less conservative thresholds. Fourth, we obtained a final threshold-weighted overlap map by taking the area under the curve of the cumulative histogram of the proportion of participants at each threshold for each region across participants. We used the following linear weighted function proposed by Seghier and Price^30^, which ensures that overlap map values range between 0 and 1, with 1 indicating an effect in a region is present in each participant at each threshold:

$${W}_{\mathrm{th}}=2\times \frac{\mathrm{th}-{z}_{\min }}{{z}_{\max }-{z}_{\min }},$$

where ${{\rm{z}}}_{{{\max }}}$ and ${z}_{\min }$ respectively correspond to the maximum and minimum *z*-value thresholds, and $\mathrm{th}$ is the given statistical threshold. We then calculated the difference between the threshold-weighted percentage overlaps between cases and the held-out HC cohort, resulting in an overlap difference map for each disorder. We performed inference on these differences using the same group-based permutation procedure described above with statistically significance defined using a threshold of ${P}_{{{\mathrm{FDR}}}} < 0.05$, two tailed.

## Mapping neural heterogeneity at the circuit level

Having investigated neural heterogeneity at the level of individual brain regions, we next evaluated whether regions showing extreme deviations in individual cases are functionally coupled to common areas, which we term circuit-level heterogeneity (Fig. 1, middle). Where prior work has identified spatial correlations between GMV difference maps and canonical functional networks estimated at the level of group means^86^, here we leverage normative modeling to move beyond such means to investigate whether person-specific GMV deviations aggregate within common functional circuits. Evidence for such aggregation offers a plausible explanation for phenotypic similarities between individuals assigned the same diagnosis, despite reports of extreme heterogeneity in deviation location at the regional level^10,12–18^. We define the functional circuitry of a brain region showing an extreme deviation as the set of brain areas that show significant FC with that region in an independent cohort of healthy individuals. To map this circuitry, we adapted elements of the lesion network mapping methodology used extensively in neurological disorders^22–24^ for use with normative modeling, as outlined in the following.

### fMRI data and processing

The functional circuitry of deviant regions was mapped using an independent cohort of 150 resting-state functional MRI (rs-fMRI) scans (71 males, age 21–35 years) from the S900 release of the Human Connectome Project^87^ (HCP). These individuals corresponded to the 150 people with the lowest total head motion, as estimated using framewise displacement^88^, from the broader set of 282 unrelated participants with the same fMRI reconstruction in the S900 release. We focused on this subsample to minimize the effects of head motion in our FC maps and to minimize computational burden due to the large number of analyses required. Mapping deviation-related functional circuity in an independent cohort is essential because we seek to understand the circuit context of deviant regions under normative conditions. Our approach aligns precisely with the logic used in lesion network mapping of neurological patients^22,23^.

The rs-fMRI data for each participant consists of four runs (rfMRI_REST1_LR, rfMRI_REST1_RL, rfMRI_REST2_LR and rfMRI_REST2_RL) acquired at two different sessions (REST1 and REST2) using two different directions of phase coding (LR: left to right, and RL: right to left). Here, we mapped deviation-related FC using the rfMRI_REST1_LR data. All rs-fMRI data were obtained using a 32-channel Siemens 3T connectome-Skyra scanner. The imaging parameters for rs-fMRI were as follows: repetition time 720 ms, echo time 33.1 ms, flip angle 52°, field of view 208 × 180 mm^2^, matrix 104 × 90, slice number 72, slice thickness 2 mm, voxel size 2 × 2 × 2 mm^3^, multiband factor 8 and 1,200 volumes. Participants were required to stay awake, relaxed and to keep their eyes open and fixed on a bright cross-hair projected on a dark background. A detailed description of the HCP data is available in previous work^87^.

The rs-fMRI data underwent the HCP’s minimal preprocessing pipeline^89^, which includes gradient-nonlinearity-induced distortion, motion correction to the single-band reference image using FMRIB’s Linear Image Registration Tool (FLIRT), echo-planar imaging (EPI) image distortion correction using TOPUP^90^; registration into standard space using a customized boundary-based-registration algorithm and single-step spline interpolation using all transforms; intensity normalization and bias field removal to resample the original EPI into Montreal Neurological Institute space. Minimal high-pass filtering was applied with a cutoff of 2,000 ms. Artifacts were then removed using independent component analysis X-noiseifier (ICA-FIX)^91^. This involves employing an automatic classifier, specifically trained for HCP data, to identify ICA components due to measurement noise, additional motion or physiological artifacts such as cardiac pulsation and respiration. Next, the volume time series were mapped into the standard CIFTI grayordinate space^87^ and smoothed to 2 mm full width at half maximum (where the smoothing was on the surface for the cortex and in volume space for subcortex). This approach results in a standard set of grayordinates in every participant that encompasses surface vertex data and subcortical volume voxel data. The mean GM signal was then removed from each grayordinate’s time series to remove residual widespread signal deflections that were not removed by ICA-FIX^92–94^. Global signal regression (GSR) has been criticized because the global signal, by construction, contains neural signal and its removal shifts the distribution of correlations so that it is approximately centered on zero^95,96^. Nonetheless, it is a highly effective denoising procedure, successfully removing contributions to fMRI signals from head motion and respiratory variations^97,98^. Invasive recordings in animal models indicate that GSR increases the correspondence between hemodynamic signals and neuronal activity^99^, and FC estimates obtained after GSR in humans are more predictive of a wide range of behavioral measures than FC estimates obtained without GSR^100^. GSR also dramatically enhances the anatomical specificity of seed-based correlation maps^101^, which is essential for precise circuit mapping. We therefore used GSR in the present analysis to enable direct comparison with the vast majority of seed-based FC studies in the literature. Alternative strategies for denoising (for example, refs. ^92,94^) may address the limitations of GSR and may provide more refined network maps.

### Mapping the functional circuitry of extreme deviations

We used seed-based FC analysis to map the functional circuitry of each region showing an extreme deviation in any individual within the clinical and HC_test_ groups. Specifically, each deviant region was used as a seed, from which the average time course was extracted for each individual in the HCP_150_ sample (Fig. 4b). These seed time courses were then correlated with all other brain grayordinates and the resulting correlation maps were subjected to Fisher’s *r*-to-*z* transformation^102^. The transformed correlation maps for each HCP_150_ participant were then aggregated using a one-way *t*-test at each grayordinate, as implemented in Permutation Analysis of Linear Models. Thresholded maps representing the functional circuitry of the seed were obtained using permutation testing (500 permutations) with a generalized Pareto approximation of the tail of the null distribution^84^ and threshold-free cluster enhancement (TFCE)^103^, run separately for the cortical and subcortical areas, with a threshold of ${P}_{{{\mathrm{FWE}}}} < 0.025$, family-wise error corrected (that is, 0.05/2 to account for cortical and subcortical analyses). This procedure was repeated for each region in which at least one extreme deviation across individuals was observed (Fig. 3b).

To quantify circuit-level overlap, we developed an approach that parallels traditional lesion network mapping methodology^22,23^ but which allows statistical inference on observed case–control differences in overlap scores at each region. Specifically, we thresholded each deviant-related FC map by applying TFCE to the grayordinate maps, as described above. We then classified an area in our 1,032-region parcellation as showing significant FC with the seed if more than 50% of its grayordinates survived the TFCE threshold. We repeated our analysis using a mapping threshold of 75% to ensure that our findings were not driven by this specific choice (Supplementary Figs. 9–11). Note, the fslr32k projection of the Schaefer cortical parcellation is missing two parcels (533 and 903, for more details, see ref. ^104^) so these were excluded from the analysis.

We mapped FC at the grayordinate level and thresholded the data in this way to leverage the superior statistical sensitivity of TFCE and to map the spatial architecture of the seed-related FC patterns more accurately. Our procedure resulted in a binary map representing the specific brain regions that comprise the functional circuitry of each seed (Fig. 3c). Then, for each individual, we took the union of the binary FC maps across that person’s set of extrema, resulting in a single map identifying areas showing significant FC to any extreme deviation expressed by the person (Fig. 3d). Separate union maps were obtained for positive and negative deviations. The overlap of these union maps was then taken for each clinical group and the HC_test_ sample, representing, for each region, the proportion of individuals in that group for whom significant deviant-related FC was identified. We refer to this image as a circuit-level overlap map (Fig. 3e).

Here we mapped the network context of GMV deviations using FC estimates of normative brain architecture mapped in an independent sample, following the approach typically used in lesion network mapping^22,23^. It is possible that patterns of FC are altered in diagnosed individuals, and that the underlying network architecture differs from normative expectations. The net effect of such alterations would be to affect coupling between deviant and other areas, and thus our normative benchmark still offers an important reference point for understanding the distributed effects of regional deviations. Further investigation of how altered patterns of FC may moderate these relationships remains an important topic for future investigation. Alternative approaches could involve mapping the network context of GMV deviations with estimates of structural connectivity obtained with diffusion MRI^105,106^ or estimates of interregional structural covariance^107^.

### Characterizing circuit-level heterogeneity

We evaluated group differences in the circuit-level overlap maps of each clinical group relative to the HC_test_ group in two ways (for a schematic overview, see Extended Data Fig. 3). First, following the analysis of regional overlaps, we computed the difference in the case and control overlap maps ($\Delta$ overlap map, Fig. 3e) and evaluated the statistical significance of these differences with respect to an empirical null distribution generated by permuting the group labels of the individual-specific FC union maps 10,000 times, with a generalized Pareto tail approximation^84^. Statistically significant differences in deviation FC overlap were identified using a threshold of ${P}_{{{\mathrm{FDR}}}} < 0.05$, two tailed (Extended Data Fig. 3a–d).

This group-based permutation test identifies regions where the overlap in deviant-related functional circuitry differs between cases and controls, but it does not preserve group differences in the total number of deviations. It therefore cannot distinguish whether any observed differences arise from a preferential aggregation of deviations within specific circuits in one group relative to the other, or whether the group differences are driven by variations in total deviation burden. Note that differences in deviation burden should not be thought of as a confound in this context, since they are an intrinsic feature of the disease that will have real phenotypic consequences. Nonetheless, it is important to determine which findings might be driven by differences in deviation burden compared with a preferential accumulation of pathology within a specific circuit for a given disorder.

To disentangle these possibilities, we considered a second, spatial null model that preserves group differences in deviation burden. Specifically, we generated an ensemble of null cortical deviation maps for each individual in the test data by spatially rotating their empirical, unthresholded deviation maps (Extended Data Fig. 3e) using Hungarian spherical spin tests^108,109^ (Extended Data Fig. 3f) and then thresholding these rotated maps in the same way as the observed data (that is, $z > |2.6|$; Extended Data Fig. 3g). The Hungarian method was used as it preserves the original values and spatial autocorrelation of the original map, which ensures that the surrogate data yields the same number of extrema as originally observed. This procedure could only be applied to the cortex, which is topologically comparable to a sphere. The choice of an appropriate null model for subcortical areas is more complicated. While model-based procedures (for example, ref. ^110^) can generate surrogates with comparable spatial autocorrelation, they require parameter tuning, can show variable fits across individuals and do not preserve the exact values of the original data, making thresholding challenging. We therefore randomly shuffled deviation values across subcortical areas before thresholding them. Note that this null model is more lenient than a spatially constrained test since the model preserves fewer features of the data. As we found no significant differences using this test, our results will not change under a more stringent, spatially constrained null model.

We then obtained individual-specific surrogate FC union maps using the same procedure described above (Extended Data Fig. 3h), and calculated surrogate within-group overlap maps (Extended Data Fig. 3i) and between-group overlap difference maps (Extended Data Fig. 3j). This procedure was repeated 10,000 times to generate a null distribution of overlap difference maps for each disorder, again using the generalized Pareto tail approximation (Extended Data Fig. 3k). Statistically significant differences were identified using a threshold of ${P}_{{{\mathrm{FDR}}}} < 0.05$, two tailed.

To summarize, group differences in circuit-level overlap were assessed with respect to two null models, one based on permutation of group labels and one based on spatial permutation of individual deviation maps. The group-based permutation test identifies regions showing differences in circuit-level overlap regardless of deviation burden. The spatial permutation test can be used to assess the degree to which such differences are driven by variations in deviation burden. More specifically, if a given brain region shows a significant difference for both tests, we have evidence to indicate that extreme deviations within the disorder preferentially aggregate within the functional circuitry of that region. If, on the other hand, a region shows a difference with the group-based permutation test but not the spatial permutation test, then the observed group differences are consistent with expectations from randomly selecting of the same number of seeds in each group, indicating that the effects observed under group-based permutation can be attributed to the fact that one group has a higher deviation burden than the other. Note that this scenario still yields important information about the extent of circuit-level heterogeneity in the disorder, since we generally expect cases to have a higher deviation burden and any resulting circuit-level overlap will still have phenotypic consequences regardless of whether such overlaps are explained by deviation burden or not. The spatial permutation test simply offers additional insights into the potential mechanisms that may drive group differences in circuit-level overlap.

### Evaluating heterogeneity across spatial scales

To examine heterogeneity across spatial scales, we subtracted the regional $\Delta$ overlap map (Fig. 2g) from the circuit-level $\Delta$ overlap map (Fig. 3f) for each clinical group separately and evaluated the magnitude of the resulting differences using the group-based permutation testing. The $\Delta$ overlap maps quantify the difference in overlap observed at each region between each diagnostic group and controls. Thus, by subtracting the two $\Delta$ overlap maps, we directly test whether case–control differences in overlap are greater at the circuit compared with the regional level.

## Mapping neural heterogeneity at the network level

The above procedure offers a means for understanding heterogeneity at the level of neural circuits that show strong FC with a deviant region. However, it is still possible for some pairs of brain regions to be affiliated with the same extended functional network despite being weakly coupled themselves (Fig. 1, right). We therefore sought to characterize neural heterogeneity at the level of the broader functional networks within which a given deviant region may be embedded.

### Assigning regions to networks

We assigned each cortical region to one of seven canonical functional cortical networks using a well-validated network parcellation^27,31^ and assigned each subcortical region to either the medial temporal lobe (amygdala and hippocampus), thalamus or basal ganglia (nucleus accumbens, globus pallidus, putamen and caudate nucleus), as done previously^28^, resulting in a total of ten distinct functional networks (Fig. 4a–c). The subcortical regions were assigned to coarse anatomical areas that were not aligned with the finely mapped cortical function networks because the correspondence between these subcortical nuclei, as parcellated here, and canonical cortical functional networks has not been extensively investigated. An alternative network assignment may influence network-level subcortical overlap values.

### Characterizing network-level heterogeneity

To characterize network-level heterogeneity, we examined the degree to which GMV deviations aggregated within each network. For each network, we estimated the proportion of individuals within each diagnostic group that showed at least one extreme deviation in a region assigned to that network, separately for positive and negative deviations (Fig. 4d). We then computed case–control differences in overlap proportions for each network (Fig. 4e).

As in the circuit-level analysis, these observed group differences in network-level overlap were evaluated with respect to two complementary null models. The first involved a group-based randomization test in which a null distribution was generated by permuting group labels 10,000 times (Extended Data Fig. 3a–d). The second null model used the 10,000 spatially rotated maps generated in the circuit-level analysis to obtain surrogate estimates of group differences in network-level overlap while preserving variation in deviation burden (Extended Data Fig. 3e–g,l–o). In both cases, the tail of the null distribution was approximated using a generalized Pareto distribution and we used a significance threshold of ${P}_{{{\mathrm{FDR}}}} < 0.05$, two tailed. As in the circuit-level analysis, these two null models allowed us to distinguish preferential network involvement from the effects of total deviation burden. Note that comparisons in overlap values between networks are complicated by differences in network size. Our analysis therefore focuses primarily on comparisons between cases and controls within networks since the control data provide a critical normative benchmark against which cases can be compared. We repeated the same analyses using a 20-network parcellation (17 cortical networks and 3 subcortical regions).

### Reporting summary

Further information on research design is available in the Nature Portfolio Reporting Summary linked to this article.

## Online content

Any methods, additional references, Nature Portfolio reporting summaries, source data, extended data, supplementary information, acknowledgements, peer review information; details of author contributions and competing interests; and statements of data and code availability are available at 10.1038/s41593-023-01404-6.

## Supplementary information

Supplementary Information Supplementary Tables 2–5 and Figs. 1–16.

Reporting Summary

Supplementary Table 1 Demographic and scanner details of the clinical and control groups.

Supplementary Data 1 Spatial overlap (%) values and *P* values (uncorrected and FDR corrected) for regional-, circuit- and network-level analyses.

## Data Availability

Summary of data availability for each dataset used in this study is described below. Autism Brain Imaging Data Exchange I (ABIDE I)^112^ and ABIDE II^113^ datasets are available through the ABIDE repository, http://fcon_1000.projects.nitrc.org/indi/abide/. The ASRB^114^ dataset is available through the ASRB repository, subject to approval of the ASRB Access Committee (https://www.neura.edu.au/discovery-portal/asrb/). First Episode Mania Study^115^, Monash Cohort^116^, Obsessive–Compulsive and Problematic Gambling study^117^, SPAINOCD^118^ and YoDA^119^ datasets are available from the principal investigators of the respective studies, subject to evaluation of the request and local ethics committee requirements. The International Multicentre persistent ADHD CollaboraTion (IMpACT-NL)^120^ and TOP15^121^ datasets are not publicly available due to privacy or ethical restrictions. Kansas Musical Depression Study (KANMDD; ds000171)^122,123^, Massachusetts Institute of Technology Autism Study (MITASD; ds000212.v1.0.0)^124,125^, Russia fMRI Depression Study (RUSMDD; ds002748.v1.0.5)^126,127^ and University of Washington ASD Study (WASHASD; ds002522.v1.0.0)^128,129^ datasets are available through the OpenNeuro repository, 10.18112/openneuro). The HCP dataset is available in the Human Connectome Project repository (https://www.humanconnectome.org/study/hcp-young-adult).

## Code availability

The PCN toolkit (version 0.16, https://github.com/amarquand/PCNtoolkit.git) was used to generate the person-specific *z*-score deviation maps. The HCP Pipelines: Seed fMRI Analysis (https://github.com/Washington-University/HCPpipelines.git) was adapted to map the regions functionally coupled to extreme deviations. Figures were generated programmatically^130^, with cortical surface renderings generated using plotSurfaceROIBoundary (https://github.com/StuartJO/plotSurfaceROIBoundary.git). All code used to produce the figures for this study can be found at https://github.com/ashlea-segal/crossdisorder_multiscale_gmv_heterogeneity.git.
