# Supplementary material for: Regional, circuit and network heterogeneity of brain abnormalities in psychiatric disorders
Source: Nat Neurosci. 2023 Aug 14;26(9):1613–29. doi: 10.1038/s41593-023-01404-6 (PMC10471501; doi:10.1038/s41593-023-01404-6)
Supplement: Supplementary file 1 — Supplementary Tables 2–5 and Figs. 1–16. [file 41593_2023_1404_MOESM1_ESM.pdf]

nature neuroscience

Article https://doi.org/10.1038/s41593-023-01404-6

Regional, circuit and network heterogeneity of brain abnormalities in psychiatric disorders

In the format provided by the authors and unedited

Supplementary Materials

Summary data:

Summary Data. Spatial overlap (%) and p-values (uncorrected and FDR-corrected) for regional-, circuit- and network-level analyses (available in separate file)

Supplementary tables:

Supplementary Table 1. Demographic and scanner details of the clinical and control groups (available in separate file)

Supplementary Table 2. Balanced classification accuracy scores (%) from the linear support vector machine within HC individuals from the CV and held-out cohorts

Supplementary Table 3. Descriptive statistics for total deviation burden summarised for each disorder

Supplementary Table 4. The degree of spatial overlap (%) of negative deviations in each network for each group

Supplementary Table 5. The degree of spatial overlap (%) of positive deviations in each network for each group

Supplementary figures:

Supplementary Figure 1. Age distributions across scan sites for each diagnostic group

Supplementary Figure 2. Age distributions across diagnostic groups for each sex

Supplementary Figure 3. Performance metrics for the normative models

Supplementary Figure 4. Regions showing greater regional overlap of extreme negative GMV deviations in controls compared to cases

Supplementary Figure 5. Spatial overlap of extreme negative GMV deviations in each group using a threshold-weighted approach

Supplementary Figure 6. Regions showing greater regional overlap of extreme negative GMV deviations in cases compared to controls, as identified using a threshold-weighted approach

Supplementary Figure 7. Regions showing greater regional overlap of extreme negative GMV deviations in controls compared to cases, as identified using a threshold-weighted approach

Supplementary Figure 8. Regions showing greater overlap in areas functionally coupled to extreme negative GMV deviations in controls compared to cases

Supplementary Figure 9. Spatial overlap in regions functionally coupled (vertex-wise threshold $p_{FWE} < 0.025$) to extreme negative deviations ($Z < -2.6$) across groups, using a parcel-mapping threshold of 75%

Supplementary Figure 10. Regions showing greater overlap in areas functionally coupled to extreme negative GMV deviations in cases compared to controls, using parcel-mapping threshold of 75%

Supplementary Figure 11. Regions showing greater overlap in areas functionally coupled to extreme negative GMV deviations in controls compared to cases, using parcel-mapping threshold of 75%

Supplementary Figure 12. Functional networks showing greater overlap in extreme negative GMV deviations in controls compared to cases

Supplementary Figure 13. Functional networks showing greater overlap in extreme negative GMV deviations in cases compared to controls using 20-network parcellation

Supplementary Figure 14. Regions showing greater regional overlap of extreme positive GMV deviations in controls compared to cases

Supplementary Figure 15. Regions showing greater overlap in areas functionally coupled to extreme positive GMV deviations in controls compared to cases

Supplementary Figure 16. Functional networks showing greater overlap in extreme positive GMV deviations in cases compared to controls

Supplementary Table 2. **Balanced classification accuracy scores (%) from the linear support vector machine within HC individuals from the CV and held-out cohorts.**

Dataset	Site	CV (HC_{train})	Held-out controls (HC_{test})
ABIDE I	CALTECH	50.00	N/A
	CMU	49.96	N/A
	LEUVEN_1	49.96	N/A
	MAX-MUN	49.87	50.00
	NYU	49.79	50.00
	PITT	49.92	N/A
	SBL	49.92	N/A
	USM	49.45	50.00
ABIDE II	BNI	49.74	50.00
	IU	49.87	N/A
ASRB	BRIS	49.83	50.00
	MELB	47.96	50.00
	PERT	49.45	N/A
	SYDN	4.91	50.00
FEMS		49.79	50.00
MON		38.01	50.45
IMPACT		44.00	51.04
KANMDD		49.96	N/A
MITASD		50.00	50.00
OCDPG		49.44	50.00
RUSMDD		50.00	50.00
SPAINOCD		45.11	49.38
TOP15		40.44	52.96
WASHASD		49.92	50.00
YoDA		47.75	49.80

*N/A = collection sites where data for HCs was < 30, therefore all HC data was included in training set.

Supplementary Table 3. **Descriptive statistics for total deviation burden summarised for each disorder.**

	**Negative extreme deviations**		**Positive extreme deviations**	
	% of participants with at least one extreme deviation	Median deviation burden, median [range]	% of participants with at least one extreme deviation	Median deviation burden, median [range]
HC_{test}	76.21	2 [0 – 72]	65.43	2 [0 – 61]
ADHD	75.82	2 [0 – 47]	69.28	1 [0 – 61]
ASD	76.73	2 [0 – 196]	75.74	3 [0 – 138]
BP	82.46	3 [0 – 95]	71.05	2 [0 – 88]
MDD	86.96	5 [0 – 108]	65.22	1 [0 – 59]
OCD	79.64	4 [0 – 59]	71.26	2 [0 – 49]
SCZ	88.51	5 [0 – 183]	65.27	1 [0 – 123]

Supplementary Table 4. **The degree of spatial overlap (%) of negative deviations in each network for each group.**

	HC_{test}	ADHD	ASD	BP	MDD	OCD	SCZ
Vis	26.77	29.41	34.16	33.77	50.93	40.12	48.3
SM	37.18	40.52	40.1	49.12	49.69	48.5	56.14
DA	24.91	39.22	39.6	42.54	48.45	37.13	47
SAL/VA	20.82	30.72	32.67	33.33	35.4	38.32	49.61
L	13.38	11.11	14.85	17.54	24.84	16.77	28.2
F	24.91	34.64	36.14	40.35	43.48	34.73	46.48
DM	35.69	36.6	35.64	47.81	54.66	47.31	53.26
MeTe	2.6	10.46	5.45	3.95	5.59	2.99	9.66
Tha	1.86	2.61	4.46	2.19	3.73	2.4	4.96
Bas	4.83	3.27	4.46	4.82	7.45	4.79	7.83

VIS	*Visual*
SM	*Somatomotor*
DA	*Dorsal attention*
SAL/VA	*Salience/ventral attention*
L	*Limbic*
F	*Frontoparietal*
DM	*Default mode*
MeTe	*Medial Temporal*
Tha	*Thalamus*
Bas	*Basal Ganglia*

Supplementary Table 5. **The degree of spatial overlap (%) of positive deviations in each network for each group.**

	HC_{test}	ADHD	ASD	BP	MDD	OCD	SCZ
Vis	26.77	27.45	37.13	33.33	22.98	35.93	24.8
SM	37.17	30.07	48.02	46.49	32.3	40.12	36.29
DA	24.91	30.72	41.58	27.63	21.74	30.54	24.02
SAL/VA	20.82	19.61	39.11	27.63	19.25	23.95	25.59
L	13.38	10.46	25.25	16.23	18.01	13.77	11.75
F	24.91	32.03	39.6	28.07	34.16	27.54	22.72
DM	35.69	33.33	45.05	35.09	42.86	34.73	31.07
MeTe	2.6	2.61	5.94	0.88	2.48	1.2	0.26
Tha	1.86	2.61	4.46	3.51	0	2.99	3.13
Bas	4.83	2.61	7.43	11.4	5.59	7.78	18.8

VIS	*Visual*
SM	*Somatomotor*
DA	*Dorsal attention*
SAL/VA	*Salience/ventral attention*
L	*Limbic*
F	*Frontoparietal*
DM	*Default mode*
MeTe	*Medial Temporal*
Tha	*Thalamus*
Bas	*Basal Ganglia*

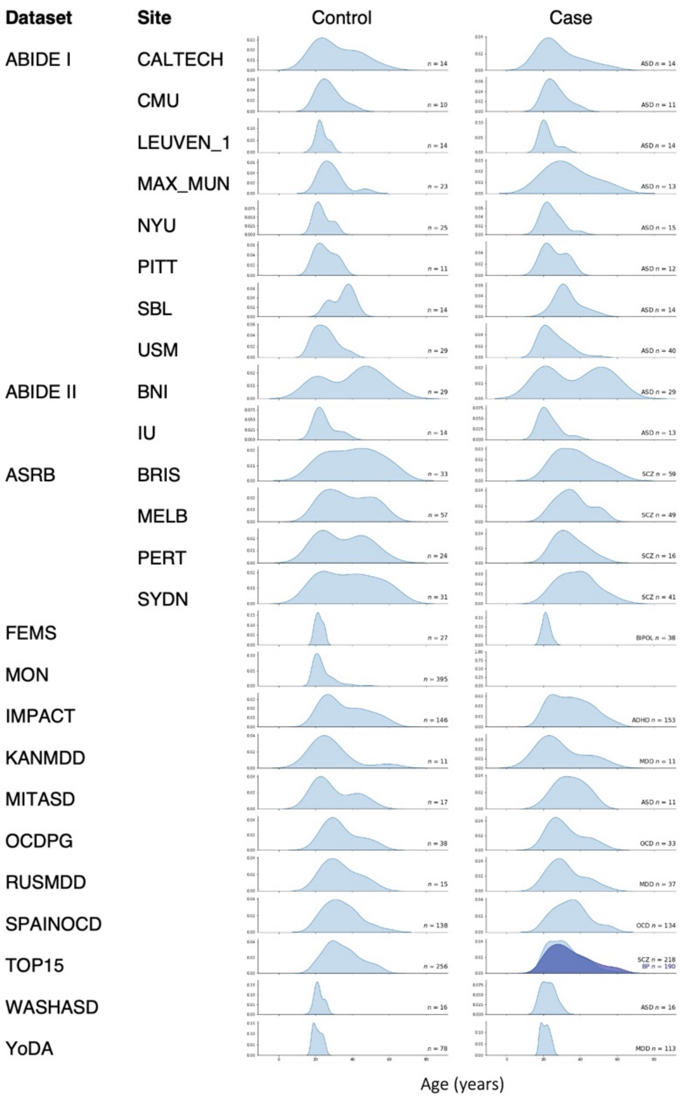

Supplementary Figure 1. **Age distribution density plots across scan sites for each diagnostic group.**

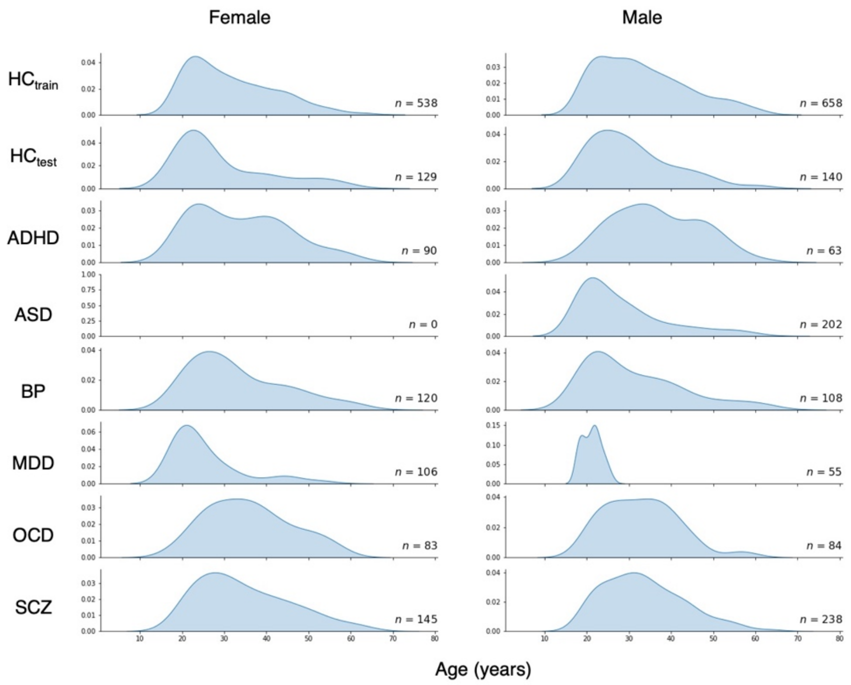

Supplementary Figure 2. **Age distribution density plots across diagnostic groups for each sex.**

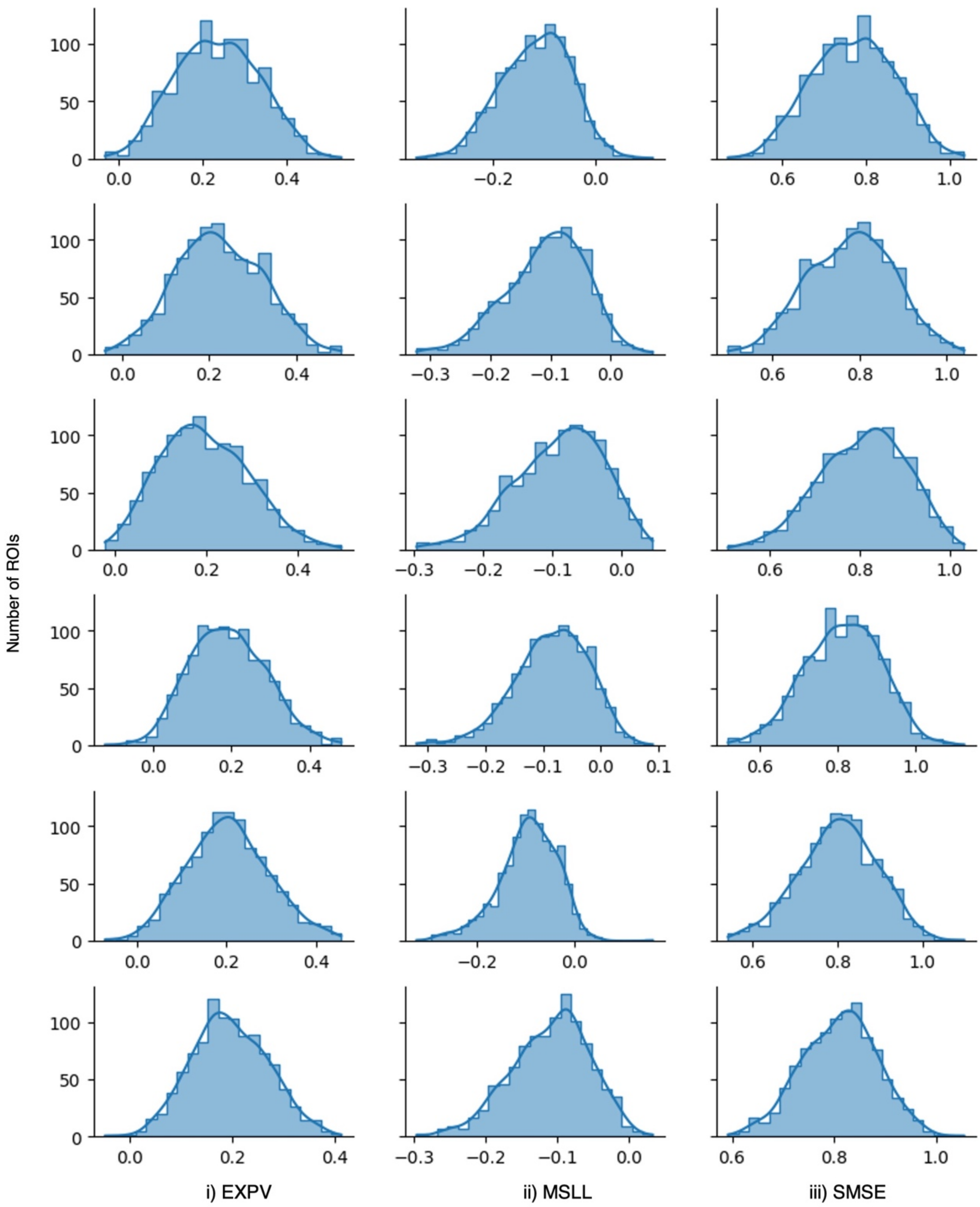

Supplementary Figure 3. **Performance metrics for the normative models.** These metrics measure the accuracy with which our normative model estimated the relationship between GMV and age, sex, and site. The distributions of i) explained variance (EXPV; higher is better), ii) Mean standardized log-loss (MSLL; lower is better), and iii) Standardized mean squared error (SMSE; lower is better) across 1000 cortical and 32 sub-cortical regions in each HC CV sample (HC_{train}). The top five rows present data from each cross-validation fold. The bottom row presents data for the test cohort.

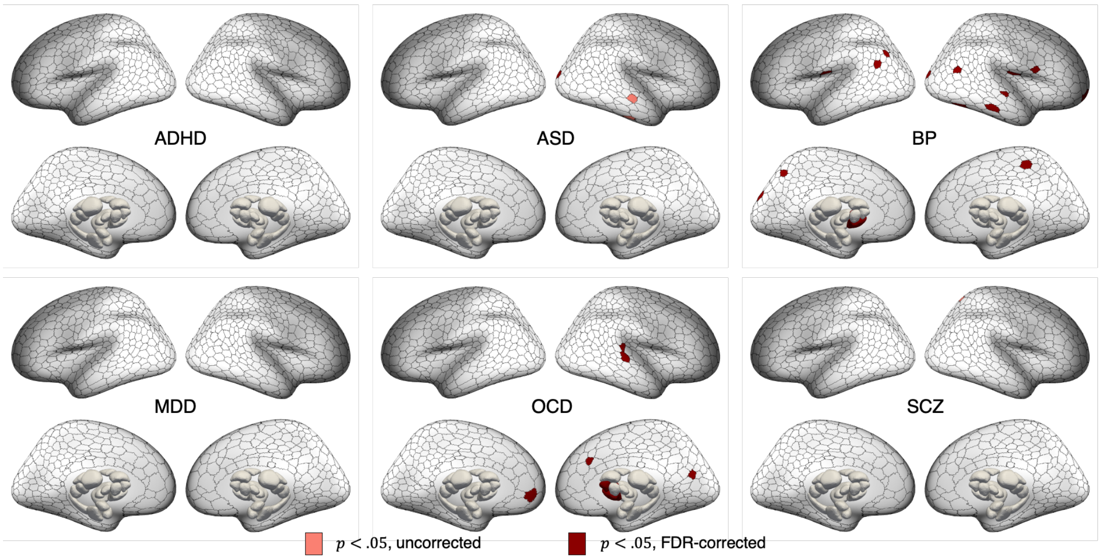

Supplementary Figure 4. **Regions showing greater regional overlap of extreme negative GMV deviations in controls compared to cases.** Statistical maps showing regions with significantly greater overlap in controls, compared to each clinical group in extreme negative deviations identified using group-based permutation tests (pink corresponds to $p_{uncorrected} < .05$, red corresponds to $p_{FDR} < .05$, two-tailed, cases<controls). Data used to generate this figure can be found in *Supplementary Data 1 (Regional_neg_thr26)*.

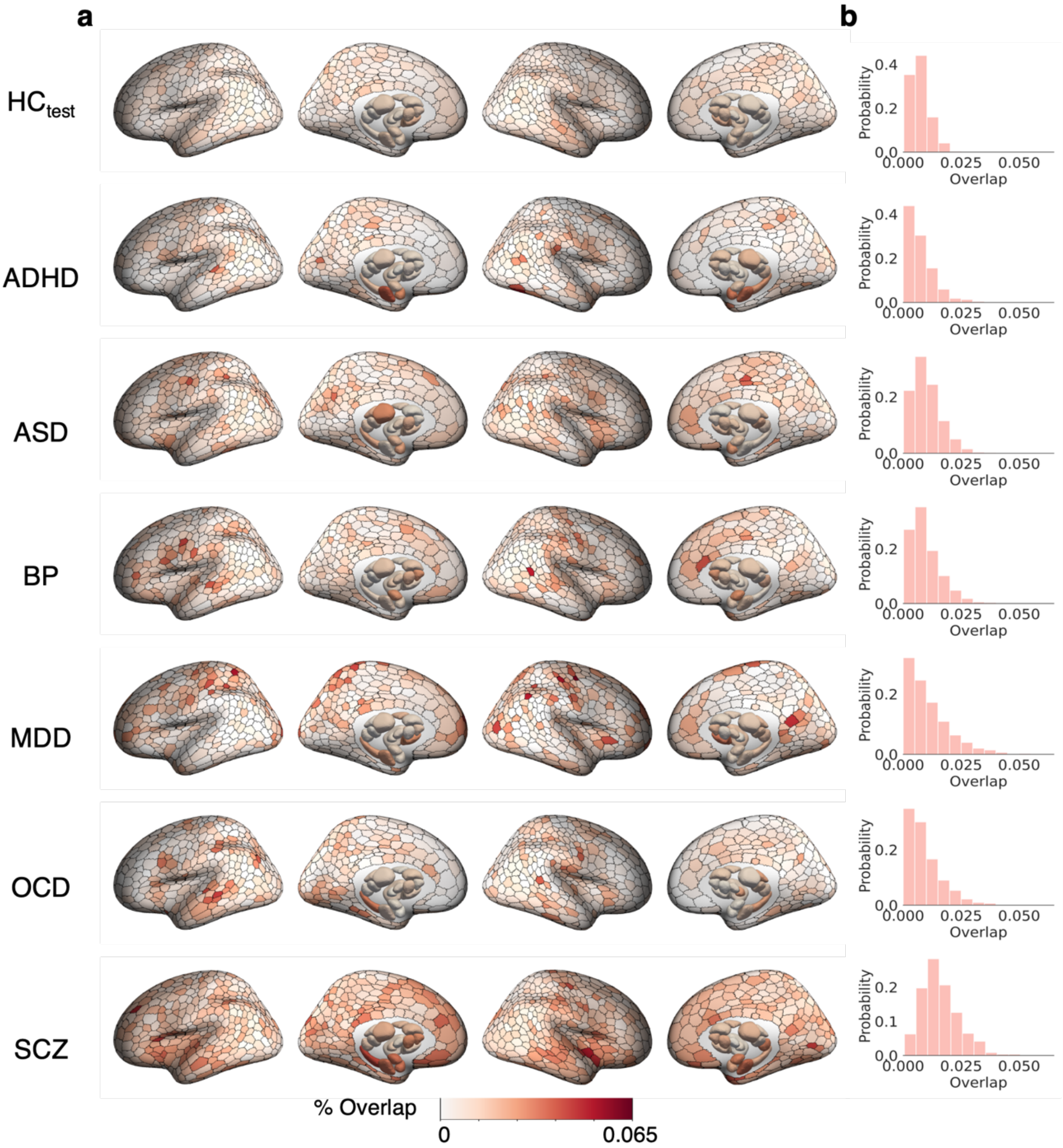

Supplementary Figure 5. **Spatial overlap of extreme negative GMV deviations in each group using a threshold-weighted approach.** a) Cortical and subcortical surface renderings showing spatial of overlap in 1032 brain regions, and b) the distribution of overlap percentages observed across all regions. Data used to generate this figure can be found in *Supplementary Data 1 (Regional_neg_thrweight)*.

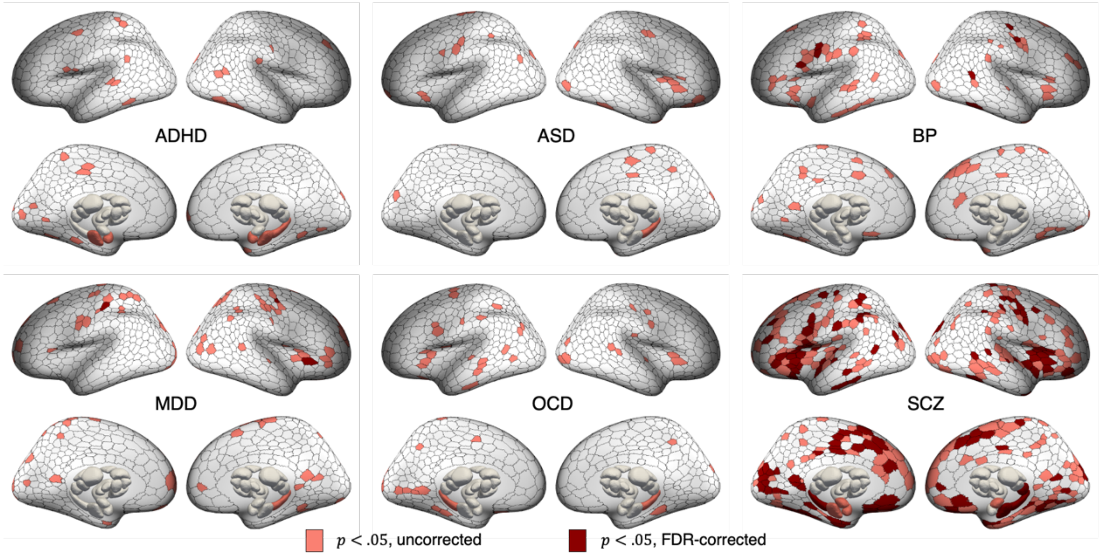

Supplementary Figure 6. **Regions showing greater regional overlap of extreme negative GMV deviations in cases compared to controls, as identified using a threshold-weighted approach.** Statistical maps showing regions with significantly greater overlap in each clinical group, compared to controls in extreme negative deviations identified using group-based permutation tests (pink corresponds to $p_{uncorrected} < .05$, red corresponds to $p_{FDR} < .05$, two-tailed, cases>controls). Data used to generate this figure can be found in *Supplementary Data 1 (Regional_neg_thrweight)*.

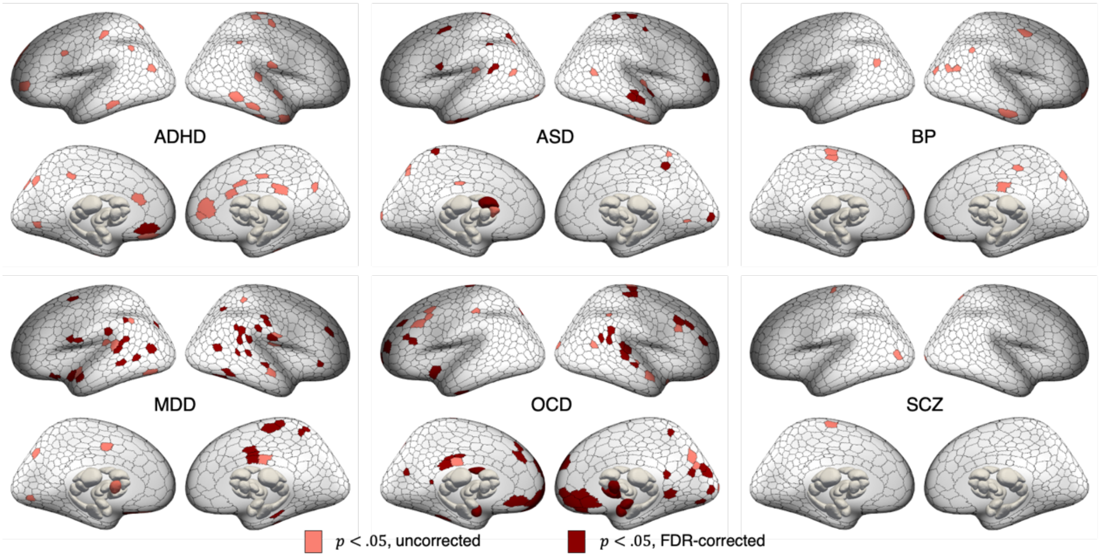

Supplementary Figure 7. **Regions showing greater regional overlap of extreme negative GMV deviations in controls compared to cases, as identified using a threshold-weighted approach.** Statistical maps showing regions with significantly greater overlap in controls, compared to each clinical group in extreme negative deviations identified using group-based permutation tests (pink corresponds to $p_{uncorrected} < .05$, red corresponds to $p_{FDR} < .05$, two-tailed, cases<controls). Data used to generate this figure can be found in *Supplementary Data 1 (Regional_neg_thrweight)*.

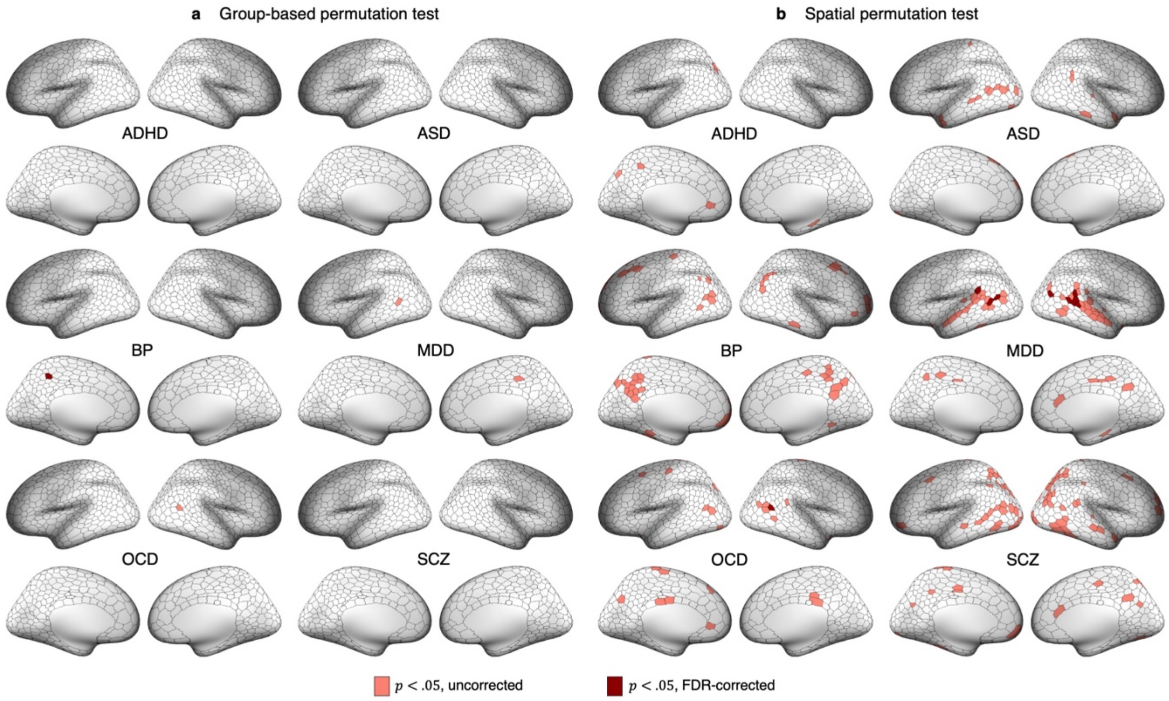

Supplementary Figure 8. **Regions showing greater overlap in areas functionally coupled to extreme negative GMV deviations in controls compared to cases.** Cortical surface renderings showing regions with significantly greater overlap in controls compared to cases in areas functionally coupled to extreme negative deviations ($p < 0.05$, two-tailed, cases<controls). (a) and (b) respectively represent significant areas identified using group-based or spatial permutation tests. The former identifies differences in overlap regardless of group differences in total deviation burden; the latter accounts for these differences and can thus reveal circuits that are preferentially impacted beyond the effects of deviation burden. Data used to generate this figure can be found in *Supplementary Data 1 (Circuit_neg_parc50)*.

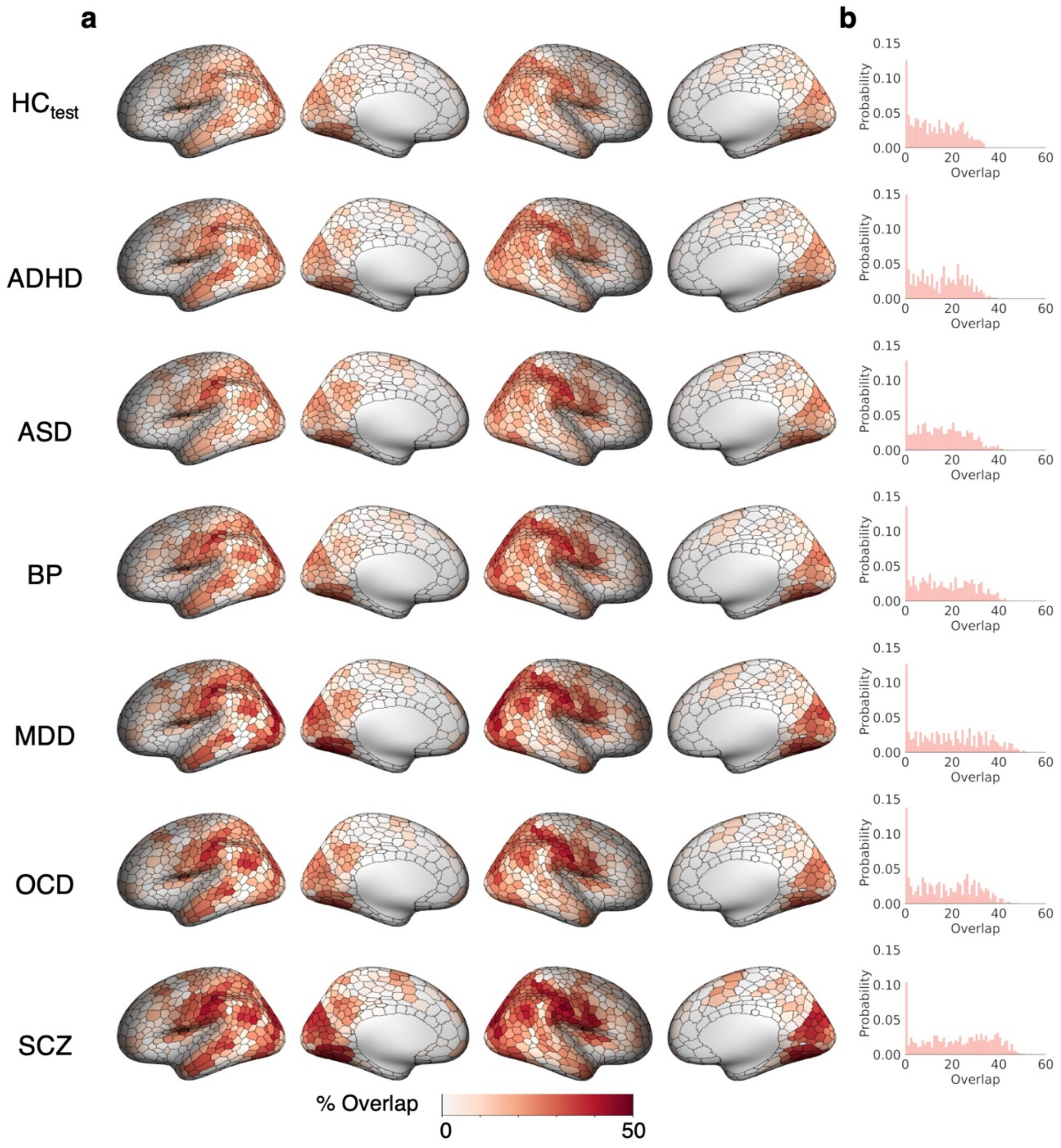

Supplementary Figure 9. **Spatial overlap in regions functionally coupled (vertex-wise threshold p_{FWE} < 0.025) to extreme negative deviations (Z < - 2.6) across groups, using a parcel-mapping threshold of 75%.** a) Cortical surface renderings showing spatial of overlap, and b) the distribution of overlap percentages observed across all regions. Data used to generate this figure can be found in *Supplementary Data 1 (Circuit_neg_parc75)*.

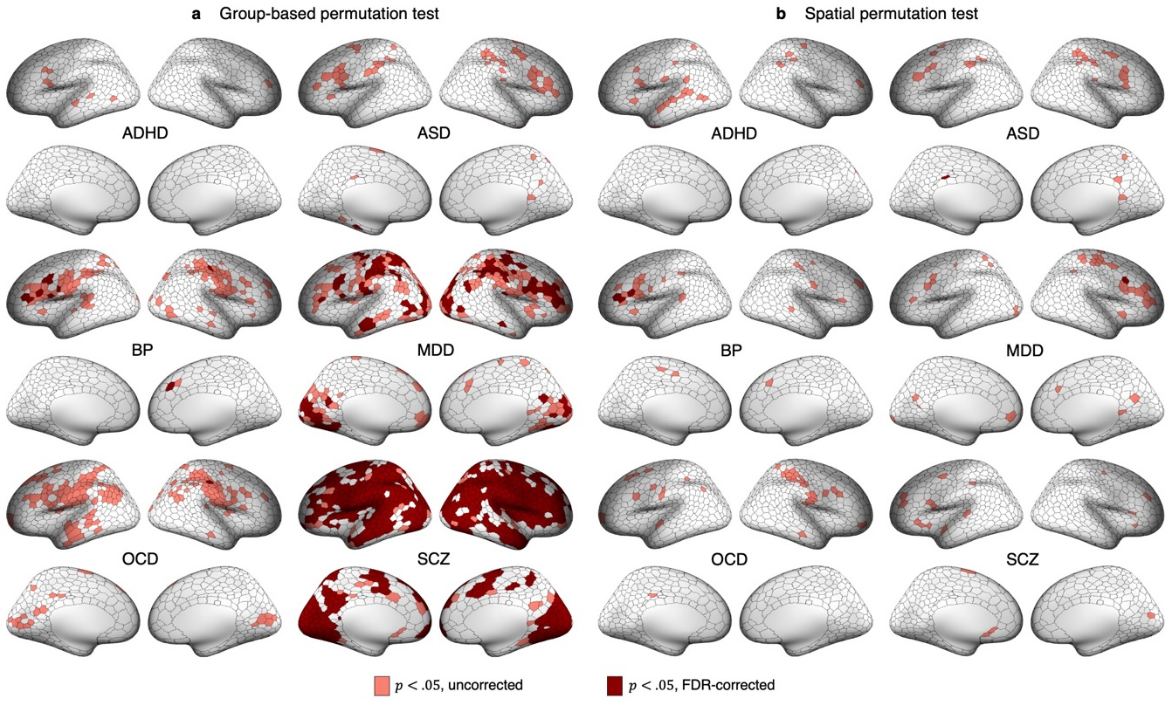

Supplementary Figure 10. **Regions showing greater overlap in areas functionally coupled to extreme negative GMV deviations in cases compared to controls, using parcel-mapping threshold of 75%.** Cortical surface renderings showing regions with significantly greater overlap in cases compared to controls in areas functionally coupled to extreme negative deviations ($p < 0.05$, two-tailed, cases>controls). (a) and (b) respectively represent significant areas identified using group-based or spatial permutation tests. The former identifies differences in overlap regardless of group differences in total deviation burden; the latter accounts for these differences and can thus reveal circuits that are preferentially impacted beyond the effects of deviation burden. Data used to generate this figure can be found in *Supplementary Data 1 (Circuit_neg_parc75)*.

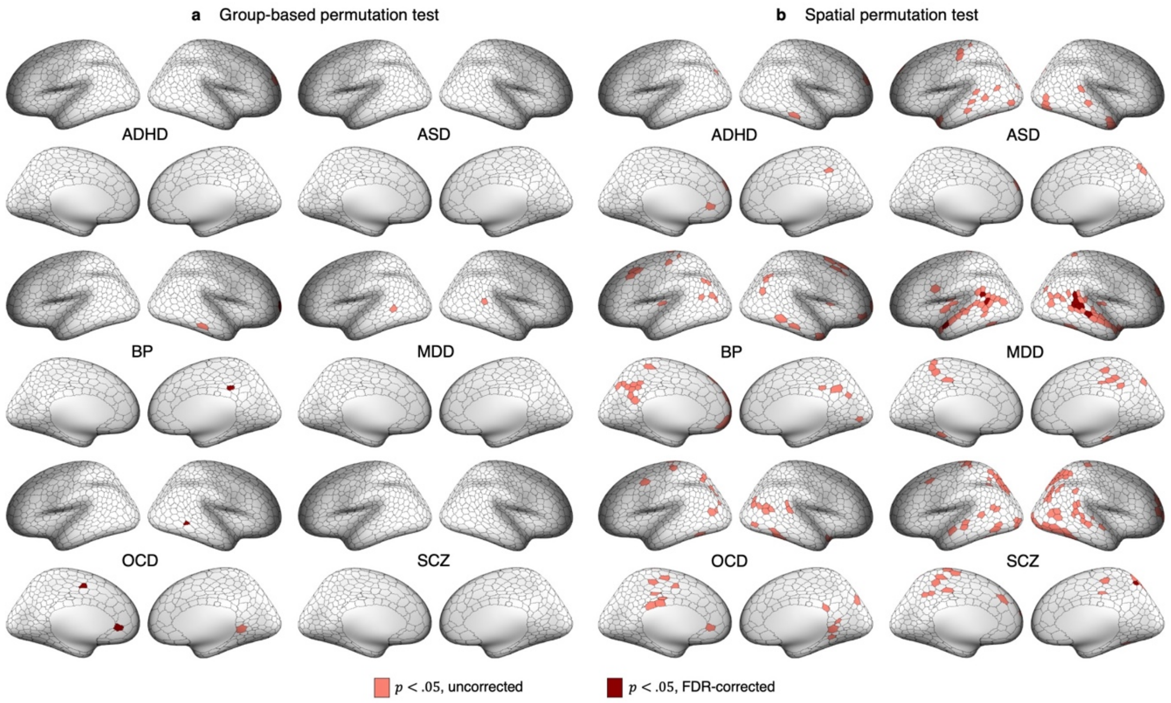

Supplementary Figure 11. **Regions showing greater overlap in areas functionally coupled to extreme negative GMV deviations in controls compared to cases, using parcel-mapping threshold of 75%.** Cortical surface renderings showing regions with significantly greater overlap in controls compared to cases in areas functionally coupled to extreme negative deviations ($p < 0.05$, two-tailed, cases<controls). (a) and (b) respectively represent significant areas identified using group-based or spatial permutation tests. The former identifies differences in overlap regardless of group differences in total deviation burden; the latter accounts for these differences and can thus reveal circuits that are preferentially impacted beyond the effects of deviation burden. Data used to generate this figure can be found in *Supplementary Data 1 (Circuit_neg_parc75)*.

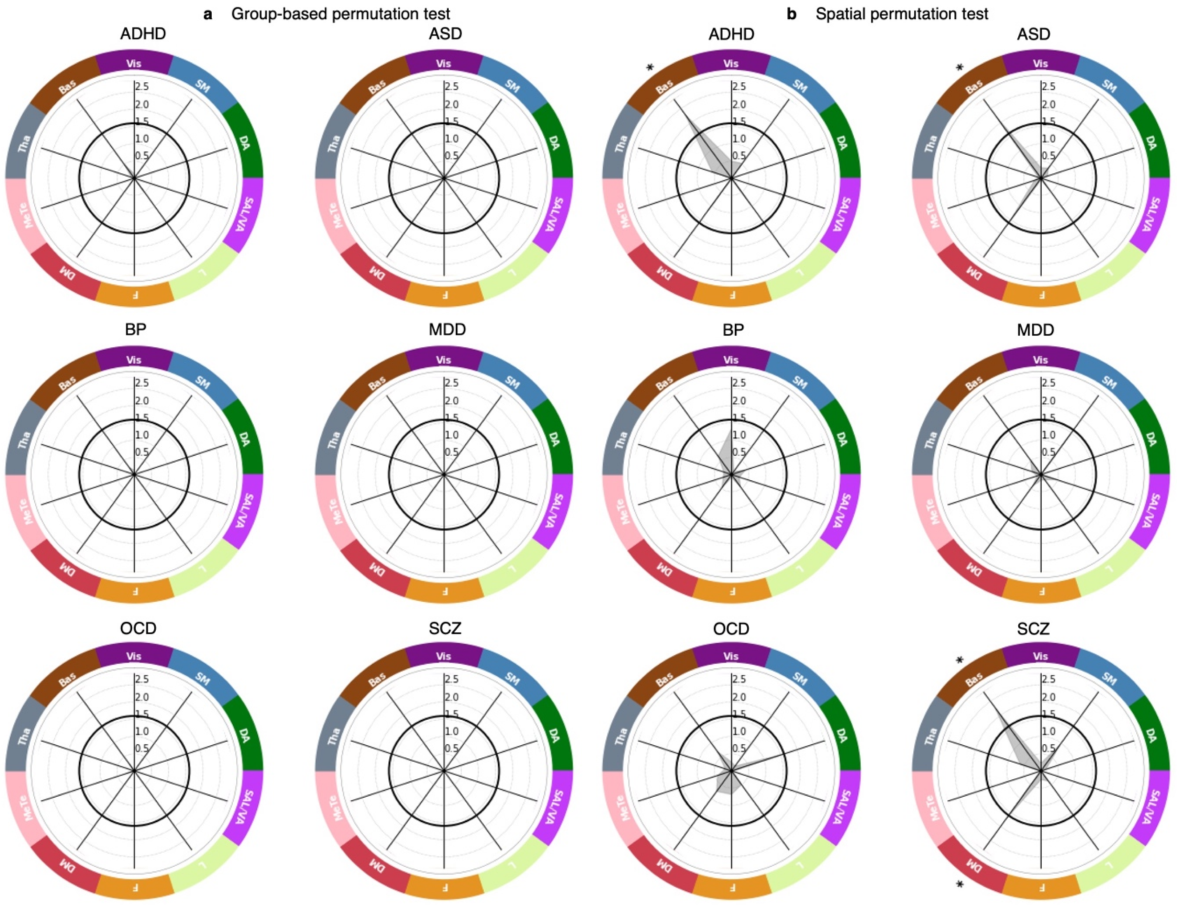

Supplementary Figure 12. **Functional networks showing greater overlap in extreme negative GMV deviations in controls compared to cases.** The network-level -$\log_{10}$ p-values associated with difference in percent overlap for extreme negative GMV deviations between each clinical group and the HC_{test} cohort. ** corresponds to $p_{FDR} < .05$, two-tailed, cases<controls, * corresponds to $p_{uncorrected} < .05$, two-tailed, cases<controls. The solid black line indicates -$\log_{10}$ p = 1.6 (*p*=0.05, two-tailed, uncorrected). (a) and (b) identify networks showing significant differences under group-based or spatial permutation testing, respectively. Data used to generate this figure can be found in *Supplementary Data 1 (Network_neg_10network)*.

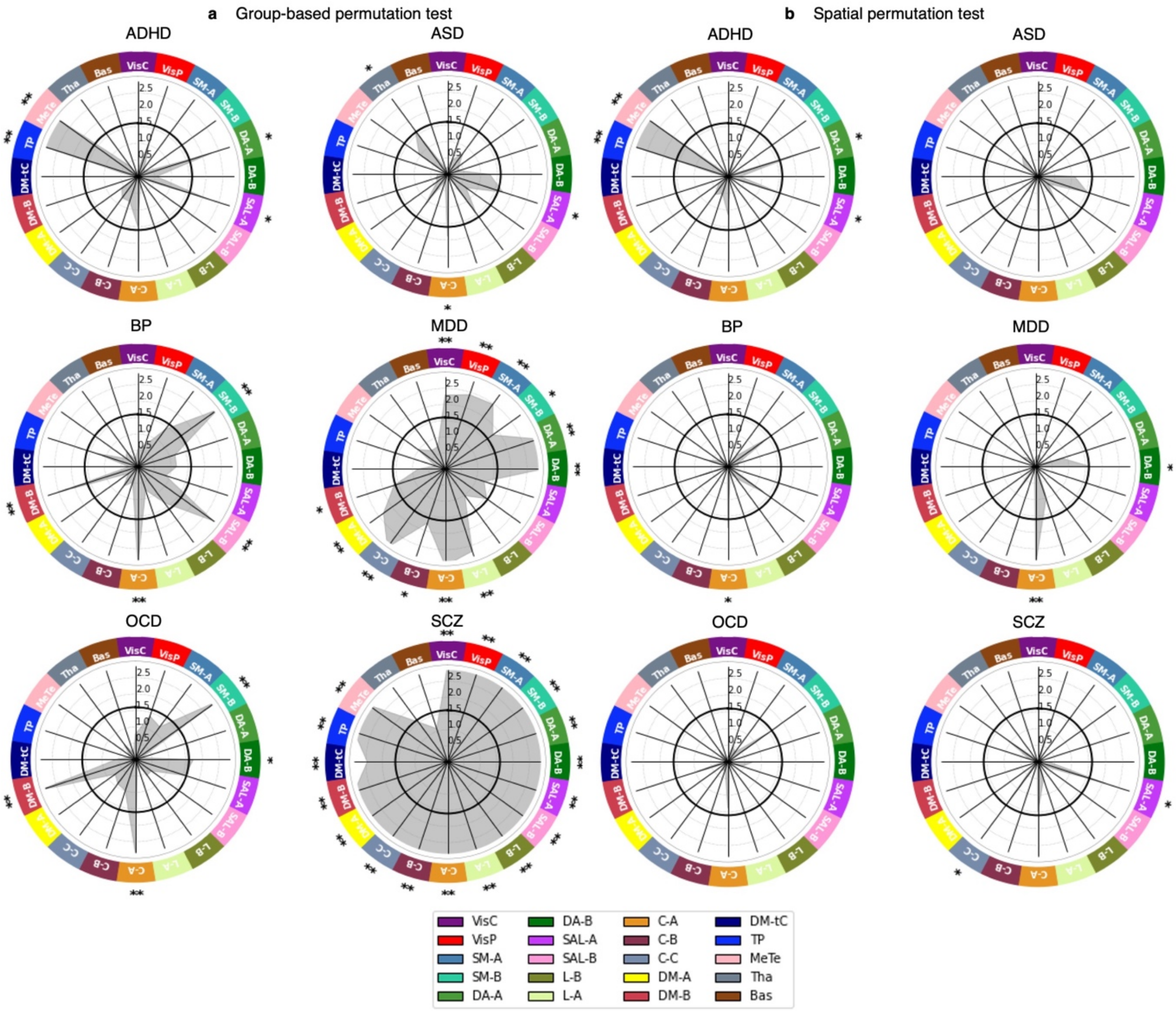

Supplementary Figure 13. **Functional networks showing greater overlap in extreme negative GMV deviations in cases compared to controls using 20-network parcellation.** The network-level -$\log_{10}$ p-values associated with difference in percent overlap for extreme negative GMV deviations between each clinical group and the HC_{test} cohort. ** corresponds to $p_{FDR} < 0.05$, two-tailed, cases>controls, * corresponds to $p_{uncorrected} < 0.05$, two-tailed, cases>controls. The solid black line indicates -$\log_{10}$ p = 1.6 (p=0.05, two-tailed, uncorrected). (a) and (b) identify networks showing significant differences under group-based or spatial permutation testing, respectively. Data used to generate this figure can be found in *Supplementary Data 1 (Network_neg_20network)*.

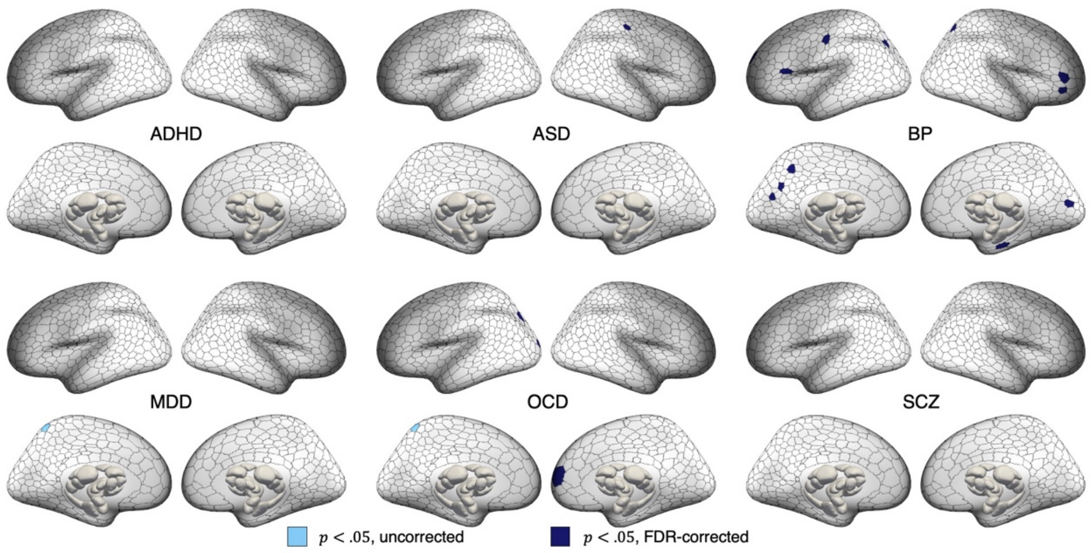

Supplementary Figure 14. **Regions showing greater regional overlap of extreme positive GMV deviations in controls compared to cases.** Statistical maps showing regions with significantly greater overlap in controls, compared to cases in extreme positive deviations identified using group-based permutation tests (light blue corresponds to $p_{uncorrected} < .05$, dark blue corresponds to $p_{FDR} < .05$, two-tailed, cases<controls). Data used to generate this figure can be found in *Supplementary Data 1 (Regional_pos_thr26)*.

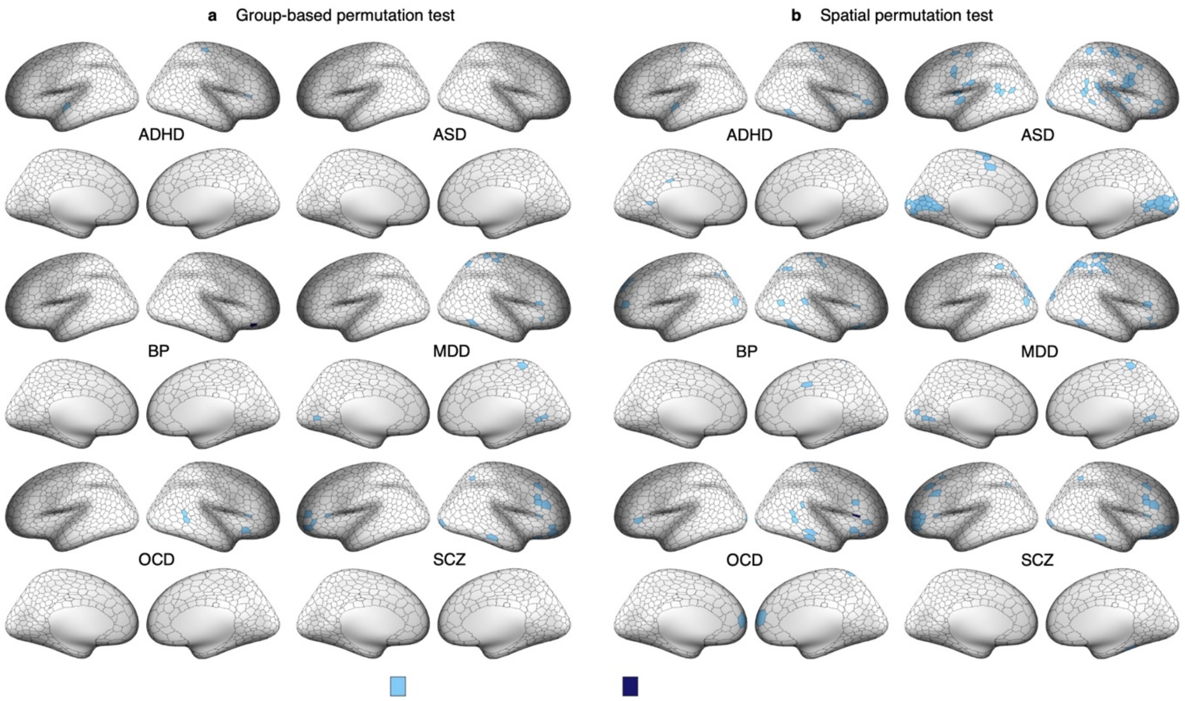

Supplementary Figure 15. **Regions showing greater overlap in areas functionally coupled to extreme positive GMV deviations in controls compared to cases.** Cortical surface renderings showing regions with significantly greater overlap in controls compared to cases in areas functionally coupled to extreme negative deviations ($p < 0.05$, two-tailed, cases<controls). (a) and (b) respectively represent significant areas identified using group-based or spatial permutation tests. The former identifies differences in overlap regardless of group differences in total deviation burden; the latter accounts for these differences and can thus reveal circuits that are preferentially impacted beyond the effects of deviation burden. Data used to generate this figure can be found in *Supplementary Data 1 (Circuit_pos_parc50)*.

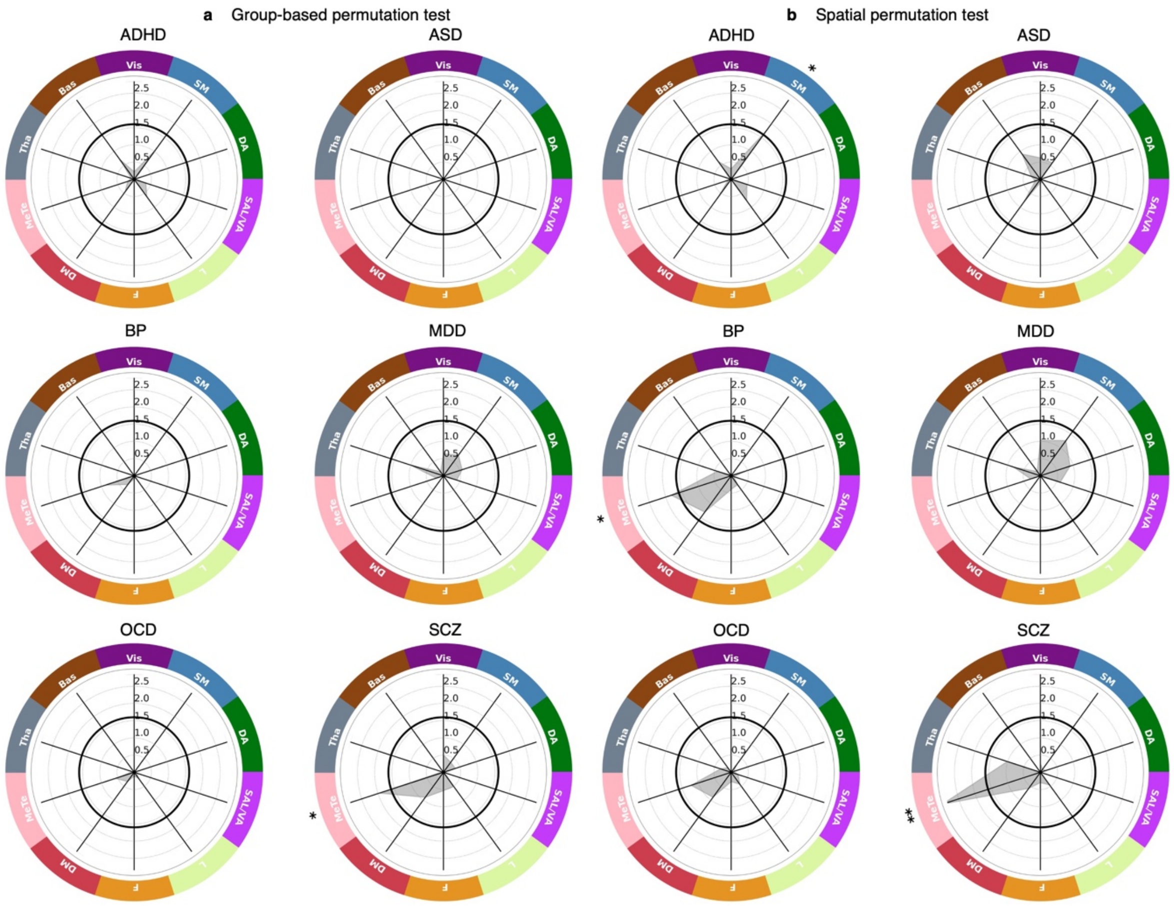

Supplementary Figure 16. **Functional networks showing greater overlap in extreme positive GMV deviations in controls compared to cases.** The network-level $-\log_{10}$ p-values associated with difference in percent overlap for extreme positive GMV deviations between each clinical group and the HC_{test} cohort. ** corresponds to $p_{FDR} < 0.05$, two-tailed, cases<controls, * corresponds to $p_{uncorrected} < 0.05$, two-tailed, cases<controls. The solid black line indicates $-\log_{10}$ p = 1.6 (p=0.05, two-tailed, uncorrected). (a) and (b) identify networks showing significant differences under group-based or spatial permutation testing, respectively. Data used to generate this figure can be found in *Supplementary Data 1 (Network_pos_10network)*.

References

1. Di Martino, A. *et al.* The autism brain imaging data exchange: Towards a large-scale evaluation of the intrinsic brain architecture in autism. *Mol. Psychiatry* **19**, 659–667 (2014).
2. Di Martino, A. *et al.* Enhancing studies of the connectome in autism using the autism brain imaging data exchange II. *Sci. Data* **4**, 1–15 (2017).
3. Loughland, C. *et al.* Australian Schizophrenia Research Bank: A database of comprehensive clinical, endophenotypic and genetic data for aetiological studies of schizophrenia. *Aust. N. Z. J. Psychiatry* **44**, 1029–1035 (2010).
4. Dandash, O. *et al.* Differential effect of quetiapine and lithium on functional connectivity of the striatum in first episode mania. *Transl. Psychiatry* **8**, (2018).
5. Sabaroedin, K. *et al.* Functional connectivity of corticostriatal circuitry and psychosis-like experiences in the general community. *Biol. Psychiatry* **86**, 16–24 (2019).
6. Hoogman, M. *et al.* Nitric oxide synthase genotype modulation of impulsivity and ventral striatal activity in adult ADHD patients and healthy comparison subjects. *Am. J. Psychiatry* **168**, 1099–1106 (2011).
7. Lepping, R. J. *et al.* 'Neural Processing of Emotional Musical and Nonmusical Stimuli in Depression'. (2018) doi:null.
8. Lepping, R. J. *et al.* Neural processing of emotional musical and nonmusical stimuli in depression. *PLoS One* **11**, 1–23 (2016).
9. Koster-Hale, J., Saxe, R., Dungan, J. & Young, L. L. Decoding moral judgments from neural representations of intentions. *Proc. Natl. Acad. Sci. U. S. A.* **110**, 5648–5653 (2013).
10. Young, L. *et al.* 'Moral judgments of intentional and accidental moral violations across Harm and Purity domains'. (2019) doi:10.18112/openneuro.ds000212.v1.0.0.
11. Parkes, L. *et al.* Transdiagnostic variations in impulsivity and compulsivity in obsessive-compulsive disorder and gambling disorder correlate with effective connectivity in cortical-striatal-thalamic-cortical circuits. *Neuroimage* **202**, 116070 (2019).
12. Bezmaternykh, D., Melnikov, M., Savelov, A. & Petrovskii, E. 'Resting state with closed eyes for patients with depression and healthy participants'. (2020) doi:10.18112/openneuro.ds002748.v1.0.5.
13. Mel'nikov, M. E. *et al.* fMRI Response of Parietal Brain Areas to Sad Facial Stimuli in Mild Depression. *Bull. Exp. Biol. Med.* **165**, 741–745 (2018).
14. Real, E. *et al.* Brain structural correlates of obsessive–compulsive disorder with and without preceding stressful life events. *World J. Biol. Psychiatry* **17**, 366–377 (2016).
15. Doan, N. T. *et al.* Distinct multivariate brain morphological patterns and their added predictive value with cognitive and polygenic risk scores in mental disorders. *NeuroImage Clin.* **15**, 719–731 (2017).
16. Kolodny, T., Schallmo, M.-P. & Murray, S. O. 'Contrast Response Functions'. (2020) doi:10.18112/openneuro.ds002522.v1.0.0.
17. Kolodny, T., Schallmo, M. P., Gerdts, J., Bernier, R. A. & Murray, S. O. Response dissociation in hierarchical cortical circuits: A unique feature of autism spectrum disorder. *J. Neurosci.* **40**, 2269–2281 (2020).
18. Davey, C. G., Cearns, M., Jamieson, A. & Harrison, B. J. Suppressed activity of the rostral anterior cingulate cortex as a biomarker for depression remission. *Psychol. Med.* (2021) doi:10.1017/S0033291721004323.
