# Supplementary material for: Regional, circuit and network heterogeneity of brain abnormalities in psychiatric disorders
Source: Nat Neurosci. 2023 Aug 14;26(9):1613–29. doi: 10.1038/s41593-023-01404-6 (PMC10471501; doi:10.1038/s41593-023-01404-6)
Supplement: Supplementary file 2 — Reporting Summary [file 41593_2023_1404_MOESM2_ESM.pdf]

nature portfolio

Corresponding author(s): Ashlea Segal, Alex Fornito

Last updated by author(s): May 30, 2023

Reporting Summary

Nature Portfolio wishes to improve the reproducibility of the work that we publish. This form provides structure for consistency and transparency in reporting. For further information on Nature Portfolio policies, see our Editorial Policies and the Editorial Policy Checklist.

Statistics

For all statistical analyses, confirm that the following items are present in the figure legend, table legend, main text, or Methods section.

n/a	Confirmed	
☐	☒	The exact sample size (n) for each experimental group/condition, given as a discrete number and unit of measurement
☐	☒	A statement on whether measurements were taken from distinct samples or whether the same sample was measured repeatedly
☐	☒	The statistical test(s) used AND whether they are one- or two-sided<br>*Only common tests should be described solely by name; describe more complex techniques in the Methods section.*
☐	☒	A description of all covariates tested
☐	☒	A description of any assumptions or corrections, such as tests of normality and adjustment for multiple comparisons
☐	☒	A full description of the statistical parameters including central tendency (e.g. means) or other basic estimates (e.g. regression coefficient) AND variation (e.g. standard deviation) or associated estimates of uncertainty (e.g. confidence intervals)
☐	☒	For null hypothesis testing, the test statistic (e.g. F, t, r) with confidence intervals, effect sizes, degrees of freedom and P value noted<br>*Give P values as exact values whenever suitable.*
☐	☒	For Bayesian analysis, information on the choice of priors and Markov chain Monte Carlo settings
☒	☐	For hierarchical and complex designs, identification of the appropriate level for tests and full reporting of outcomes
☐	☒	Estimates of effect sizes (e.g. Cohen's d, Pearson's r), indicating how they were calculated

Our web collection on statistics for biologists contains articles on many of the points above.

Software and code

Policy information about availability of computer code

Data collection

No software was used for data collection. Neuroimaging and behavioural data were from existing datasets (detailed below) whose acquisition's are presented in detail in previous work.

Data analysis

T1-weighted MRIs were preprocessed using the default settings of the Computational Anatomy Toolbox (CAT12, r113, http://dbm.neuro.uni-jena.de/cat/) and Statistical Parametric Mapping (SPM12, http://www.fil.ion.ucl.ac.uk/spm/software/spm12), using MATLAB v9.8. This involved using Diffeomorphic Anatomical Registration Exponentiated Lie Algebra (DARTEL) to normalise the segmented scans. Example code and preprocessing report can be found here: https://github.com/ashlea-segal/multiscale-heterogeneity-brain-abnormalities

rs-fMRI HCP data was preprocessed using the HCP minimal preprocessing pipeline (Glasser et al., 2013, Neuroimage). This included FSL FLIRT, TOPUP, ICA-FIX, and HCP Connectome Workbench (v1.2.3). HCPpipelines TaskfMRIAnalysis was adapted for the rs-fMRI seed-based analysis. Code can be found here: https://github.com/Washington-University/HCPpipelines.

Normative Modelling Software: PCNtookit (version= 0.16, https://github.com/amarquand/PCNtoolkit) to generate person-specific z-score deviation maps.

Permutation Analysis of Linear Models software package (PALM alpha116, https://github.com/andersonwinkler/PALM) for statistical inference

Specific packages used within Anaconda virtual environment and code to generate figures can be found here: https://github.com/ashlea-segal/multiscale-heterogeneity-brain-abnormalities

For manuscripts utilizing custom algorithms or software that are central to the research but not yet described in published literature, software must be made available to editors and reviewers. We strongly encourage code deposition in a community repository (e.g. GitHub). See the Nature Portfolio guidelines for submitting code & software for further information.

Data

Policy information about availability of data

All manuscripts must include a data availability statement. This statement should provide the following information, where applicable:

- Accession codes, unique identifiers, or web links for publicly available datasets
- A description of any restrictions on data availability
- For clinical datasets or third party data, please ensure that the statement adheres to our policy

This study used neuroimaging data collected across multiple independent datasets, with varying levels of data accessibility. Below summarizes the availability of each dataset.

Autism Brain Imaging Data Exchange I (ABIDE I) and ABIDE II data used in this study came from the ABIDE 1000 Functional Connectomes Project repository, http://fcon_1000.projects.nitrc.org/indi/abide/. Data usage is unrestricted for non-commercial research purposes. As per INDI protocol, users must register with the NITRC and 1000 Functional Connectomes Project to gain access.

The Australian Schizophrenia Research Bank (ASRB) is a medical research database and storage facility that links clinical and neuropsychological information, blood samples and strcutral MRI brain scans from people with schizophrenia and healthy non-psychiatric controls. Data is available in the ASRB repository, subject to approval of the ASRB Access Committee https://www.neura.edu.au/discovery-portal/asrb/

First Episode Mania Study (FEMS) - Available from the principal investigator of the study, subject to local ethics committee requirements.
Monash Cohort (MON) - Available from the principal investigator of the study, subject to local ethics committee requirements

International Multi-centre persistent ADHD CollaboraTion (IMpACT-NL) - Not publicly available due to privacy or ethical restrictions.

OpenNeuro - Kansas Musical Depression Study (KANMDD) - Available in the OpenNeuro repository, https://openneuro.org/datasets/ds000171

OpenNeuro - Massachusetts Institute of Technology Autism Study (MITASD) - Available in the OpenNeuro repository, https://doi.org/10.18112/openneuro.ds000212.v1.0.0

Obsessive-compulsive and problematic gambling study (OCDPG) - Available from the principal investigator of the study, subject to local ethics committee requirements

OpenNeuro - Russia fMRI Depression Study (RUSMDD) - Available in the OpenNeuro repository, https://doi.org/10.18112/openneuro.ds002748.v1.0.5

SPAINOCD - Available from the principal investigator of the dataset, subject to local ethics committee requirements

TOP15 - The data are not publicly available due to privacy or ethical restrictions.

OpenNeuro – University of Washington ASD Study (WASHASD) - Available in the OpenNeuro repository, https://doi.org/10.18112/openneuro.ds002522.v1.0.0

YoDA - Available from the principal investigator of the study, subject to local ethics committee requirements

Human Connectome Project (HCP) - Available in the Human Connectome Project repository, https://www.humanconnectome.org/study/hcp-young-adult. Users must agree to data use terms for the HCP before being allowed access to the data and ConnectomeDB, details are provided at https://www.humanconnectome.org/study/hcp-young-adult/data-use-terms.

No new data were collected for this manuscript. Across all datasets, we did not use any specific software for downloading the data. For details on data collection for each data, please see relevant references in Supplementary Table 1.

Field-specific reporting

Please select the one below that is the best fit for your research. If you are not sure, read the appropriate sections before making your selection.

☒ Life sciences ☐ Behavioural & social sciences ☐ Ecological, evolutionary & environmental sciences

For a reference copy of the document with all sections, see nature.com/documents/nr-reporting-summary-flat.pdf

Life sciences study design

All studies must disclose on these points even when the disclosure is negative.

Sample size	No sample size calculations were performed. This projects uses open-access data and data made available through collaborators. Combined, the sample consists of 1465 healthy controls (HC; 54.47% male) and 1294 cases, taken from 14 different studies and 25 different scan sites. The clinical sample comprised 202 individuals with ASD (100% male), 153 individuals with ADHD (41.18% male), 228 individuals with BP (47.37% male), 161 individuals with MDD (34.16% male), 167 individuals with OCD (50.30% male), and 383 participants with SCZ (62.14% male). See manuscript for extended discussion.

HCP data was used because of the large number of subjects and the relatively equal male/female balance. For HCP data, only unrelated subjects were included.

Data exclusions	All T1-weighted images were visually inspected and evaluated for the presence of artifacts, resulting in the exclusion of 53 images with gross artifacts or abnormalities. Next, we used the Computational Anatomy Toolbox (CAT12 r113, http://dbm.neuro.unijena.de/cat/) to generate a weighted overall image quality rating (IQR) for every scan. This metric combines ratings of basic image properties, including the level of noise and geometric distortions, into a single score that quantifies the overall image quality of a participant's T1-weighted scan (see here for more information www.neuro.uni-jena.de/cat/index.html#QA). On this metric, lower scores denote higher image quality. As per previous work21, we excluded 153 images with an IQR > 2.8. An additional 63 images were excluded due to a failure of our processing pipeline. Collectively, this quality assurance process resulted in the exclusion of a total of 269 scans.<br>Following this quality control protocol, we performed a series of additional quality control checks and exclusions for our analysis. Specifically, we excluded participants if they were below 18 years or above 64 years of age (N=346); did not have the necessary clinical data (clinical diagnosis or, for healthy controls, absence of any clinical diagnosis) or demographic information (age, sex and scanner site, N=53); if their T1-weighted structural magnetic resonance imaging (MRI) scan did not survive our stringent manual and automated quality control procedure, as explained below (N=269); or if the data came from a site with less than 10 individuals in the same group and sex (described in the Normative model section, below; N=217). Our final sample available for analysis thus comprised 1465 HCs and 1294 cases.
Replication	We have included numerous quality control checks to verify the stability of our findings, including:<br>• Cross-validation of the normative models to assess model generalisability<br>• Multiple linear support vector machines to evaluate the model's efficacy in partitioning site-related variance<br>• Pearson's correlation between number of extreme deviations and image quality to assess whether individual variations in scan quality affected the deviation zmaps<br>• Threshold-weighted analysis of deviation mapping to show that our results do not depend on the choice of a specific threshold for defining deviations<br>• The inclusion of a held-out control group for all analyses to establish a normative benchmark for assessing % overlap in the clinical groups<br>• Mapping circuit-level overlap using two mapping thresholds (50% and 75%) to ensure our findings were not driven by this specific choice<br>• Statistical inference at the circuit- and network- level using two different null models to disentangle whether any differences arise from a preferential aggregation of deviations or whether group differences are driven by variations in total deviation burden
Randomization	Randomization was not performed because participants were not placed into experimental groups - case and control groups are defined based on diagnosis. Participants without a diagnosis of a psychiatric disorder were in the control group. Participants diagnosed with a psychiatric disorder were placed in the clinical group, which was further divided by diagnosis (ADHD, ASD, BP, OCD, MDD, SCZ).
Blinding	Blinding is not relevant to this study because participants were not placed into experimental groups.

Reporting for specific materials, systems and methods

We require information from authors about some types of materials, experimental systems and methods used in many studies. Here, indicate whether each material, system or method listed is relevant to your study. If you are not sure if a list item applies to your research, read the appropriate section before selecting a response.

Materials & experimental systems

n/a	Involved in the study	
☒	☐	Antibodies
☒	☐	Eukaryotic cell lines
☒	☐	Palaeontology and archaeology
☒	☐	Animals and other organisms
☐	☒	Human research participants
☒	☐	Clinical data
☒	☐	Dual use research of concern

Methods

n/a	Involved in the study	
☒	☐	ChIP-seq
☒	☐	Flow cytometry
☐	☒	MRI-based neuroimaging

Human research participants

Policy information about studies involving human research participants

Population characteristics	See above in Life sciences study design: Sample size. All participants were aged between 18-64 years old.
Recruitment	See the relevant reference for each dataset for specific details of each dataset. In general, the sample was collected through a range of different recruitment resources including media advertisements, inpatient, outpatient and community mental health service providers, non-government organizations, rehabilitation services, and cold telephoning using the electoral rolls.
Ethics oversight	The study was approved by the local ethics committee of each dataset, and written informed consent was obtained from each participant. This study was approved by the Monash University Research Ethics Committee (Project ID: 23534).

Note that full information on the approval of the study protocol must also be provided in the manuscript.

Magnetic resonance imaging

Experimental design

Design type	resting-state fMRI, structural (VBM) MRI
Design specifications	HCP: The rs-fMRI data for each subject consists of four runs (rfMRI_REST1_LR, rfMRI_REST1_RL, rfMRI_REST2_LR, rfMRI_REST2_RL) acquired at two different sessions (REST1 and REST2) using two different directions of phase coding (LR: left to right, and RL: right to left).
Behavioral performance measures	Behavioral performance measures were not applicable in this study

Acquisition

Imaging type(s)	resting-state fMRI, structural (VBM) MRI
Field strength	Described in Supplementary Table 1 for each dataset.
Sequence & imaging parameters	Described in Supplementary Table 1 for each dataset for structural data.<br><br>HCP rs-fMRI data were obtained using a 32-channel Siemens 3T connectome-Skyra scanner. The imaging parameters for rs-fMRI were as follows: repetition time (TR)=720 ms, echo time (TE)=33.1 ms, flip angle (FA)=52°, field of view (FOV)=208×180 mm2, matrix=104×90, slice number=72, slice thickness=2 mm, voxel size=2×2×2 mm3, multiband factor=8, and 1200 volumes.
Area of acquisition	Whole brain

Diffusion MRI ☐ Used ☒ Not used

Preprocessing

Preprocessing software

Preprocessing of the structural and functional data was done using a suite of tools.

Structural preprocessing
GMV was estimated using the CAT12 VBM pipeline, which is included as an extension of Statistical Parametric Mapping software (SPM12, http://www.fil.ion.ucl.ac.uk/spm/software/spm12) in MATLAB v9.8 using the default settings. Briefly, the T1-weighted images were first corrected for intensity nonuniformities, and segmented into GM, white matter (WM), and cerebrospinal fluid (CSF) tissue probability maps.
Using the high- dimensional Diffeomorphic Anatomical Registration Exponentiated Lie Algebra (DARTEL), the segmented scans were normalized into standard Montreal Neurological Institute (MNI) space. Lastly, the images were bias-field corrected and modulated by the linear and nonlinear components of the Jacobian determinant obtained from the DARTEL deformation fields to obtain voxel-wise estimates of GMV.

Functional preprocessing
The rs-fMRI data used underwent the HCP's minimal pre-processing pipeline, which includes gradient-nonlinearity-induced distortion, motion correction to the single-band reference image using FLIRT, EPI image distortion correction using TOPUP, registration into standard space using a customized boundary-based-registration (BBR) algorithm, and single step spline interpolation using all transforms, intensity normalization and bias field removal to resample the original EPI into MNI space. Minimal high pass filtering was applied with a cutoff of 2000ms. Artifacts were then removed using ICA-FIX. This involves employing an automatic classifier, specifically trained for HCP data, to identify ICA components due to measurement noise, additional motion or physiological artifacts like cardiac pulsation and respiration. Next, the volume timeseries were mapped into the standard CIFTI grayordinate space and smoothed to 2mm FWHM (where the smoothing was on the surface for the cortex and in volume space for subcortex). This results in a standard set of grayordinates in every subject, with surface vertex data and subcortical volume voxel data. The mean GM signal was then removed each grayordinate's time series to remove residual widespread signal deflections that are not removed by ICA-FIX

Normalization	See above
Normalization template	MNI Standard Space
Noise and artifact removal	See above
Volume censoring	See above

Statistical modeling & inference

Model type and settings	Mass univariate across multiple brain scales (regional, circuit, network; see manuscript)
Effect(s) tested	As the primary aim of this study was to investigate anatomically heterogeneous brain deviations across multiple brain scales (regional, circuit, network) across six psychiatric disorders, multiple effects were examined. The main effect - observed group

differences between each patient group and controls was evaluated with respect to two complementary nulls detailed in Supplementary Figure 6.

Specify type of analysis: ☐ Whole brain ☐ ROI-based ☒ Both

Anatomical location(s): Parcel-level analyses utilised the Schaefer et al., 2018 Cerebral Cortex 1000 region parcellation for cortical regions, and the Tian et al Nature Neuroscience 32 region parcellation for subcortical regions.

Statistic type for inference (See Eklund et al. 2016): Non-parametric inference was used across multiple neuroanatomical scales including regions of interest, and networks (see manuscript)

Correction: Results were presented at $p<0.05$ uncorrected and following False Discovery Rate (FDR, q=0.05) correction.

Models & analysis

n/a	Involved in the study
☐	☒ Functional and/or effective connectivity
☒	☐ Graph analysis
☒	☐ Multivariate modeling or predictive analysis

Functional and/or effective connectivity: Pearson correlation
